# ABCC2 induces metabolic vulnerability and cellular ferroptosis via enhanced glutathione efflux in gastric cancer

**DOI:** 10.1002/ctm2.1754

**Published:** 2024-08-02

**Authors:** Yiding Wang, Xuejun Gan, Xiaojing Cheng, Yongning Jia, Gangjian Wang, Xiaohuan Tang, Hong Du, Xiaomei Li, Xijuan Liu, Xiaofang Xing, Jiafu Ji, Ziyu Li

**Affiliations:** ^1^ Department of Gastrointestinal Cancer Translational Research Key Laboratory of Carcinogenesis and Translational Research (Ministry of Education) Peking University Cancer Hospital & Institute Beijing P.R. China; ^2^ Department of Gastrointestinal Cancer Center Ward I Peking University Cancer Hospital & Institute Beijing P.R. China; ^3^ Department of Central Laboratory Key Laboratory of Carcinogenesis and Translational Research (Ministry of Education/Beijing) Peking University Cancer Hospital & Institute Beijing China

**Keywords:** ABCC2, amino acid metabolism, ferroptosis, gastric cancer, glutathione

## Abstract

**Background:**

Although it is traditionally believed that ATP binding cassette subfamily C member 2 (ABCC2) is a multidrug resistance‐associated protein correlated with a worse prognosis, our previous and several other studies demonstrated the contrary to be true in gastric cancer (GC). We aim to explore the underlying mechanism of this discovery.

**Methods:**

Our study utilized whole‐exome sequencing (WES), RNA sequencing, and droplet digital PCR (ddPCR) analysis of 80 gastric cancer samples, along with comprehensive immunohistochemical (IHC) analysis of 1044 human GC tissue samples.By utilizing CRISPRCas9 to genetically modify cell lines with the ABCC2‐24C > T (rs717620) point mutation and conducting dual‐luciferase reporter assays, we identified that transcription factors SOX9 and ETS1 serve as negative regulators of ABCC2 expression. Seahorse assay and mass spectrometry were used to discover altered metabolic patterns. Gain and loss‐of‐function experiments in GC cell lines and preclinical models were carried out to validate ABCC2 biological function.

**Results:**

ABCC2 high expression correlated with better prognosis, and rs717620 can influence ABCC2 expression by disrupting the binding of ETS1 and SOX9. Gain and loss‐of‐function experiments in GC cell lines demonstrated amino acid deprivation reduces proliferation, migration, and drug resistance in ABCC2‐high GC cells. ABCC2 leads to reduced intracellular amino acid pools and disruption of cellular energy metabolism. This phenomenon depended on ABCC2‐mediated GSH extrusion, resulting in alterations in redox status, thereby increasing the cell's susceptibility to ferroptosis. Furthermore, patient‐derived organoids and patient‐derived tumor‐like cell clusters were used to observe impact of ABCC2 on therapeutic effect. In the xenograft model with high ABCC2 expression, we observed that constricting amino acid intake in conjunction with GPX4 inactivation resulted in notable tumor regression.

**Conclusions:**

Our findings demonstrate a significant role of ABCC2 in amino acid metabolism and ferroptosis by mediating GSH efflux in GC. This discovery underlines the potential of combining multiple ferroptosis targets as a promising therapeutic strategy for GC with high ABCC2 expression.

**Highlights:**

- ABCC2 plays a crucial role in inducing metabolic vulnerability and ferroptosis in gastric cancer through enhanced glutathione efflux.
- The ABCC2 24C > T polymorphism is a key factor influencing its expression.
- These results highlight the potential of ABCC2 as a predictive biomarker and therapeutic target in gastric cancer.

## Graphical abstract

(1) ABCC2 expression is increased by the ABCC2‐24C > T polymorphism, improving clinical outcomes in gastric cancer. (2) High ABCC2 expression disrupts intracellular amino acid pools via glutathione efflux, altering redox status and enhancing susceptibility to ferroptosis. (3) Combining amino acid restriction with ferroptosis inducers significantly reduces tumour growth in high ABCC2 expression models.

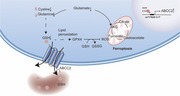

## 1 BACKGROUND

Gastric cancer (GC) ranks as the fifth most prevalent malignancy globally and is the third leading cause of cancer‐related mortality, with 1 089 103 new cases diagnosed and approximately 768 793 deaths reported worldwide in 2020.^1^, ^2^, ^3^ Common therapies for gastric cancer include 5‐fluorouracil (5‐Fu) and platinum‐based treatments, which elicit varying responses among patients.^4^, ^5^ Given its complex and dynamic molecular background, gastric cancer is a highly heterogeneous malignancy. Therefore, elucidating the molecular mechanisms driving the progression of gastric cancer is essential for developing novel and effective treatment strategies.

In a previous study, our team examined genetic variations in several genes, including MTHFR, DPYD, UMPS, ABCB1, ABCC2, GSTP1, ERCC1, and XRCC1, in a group of patients with gastric cancer who were undergoing neoadjuvant chemotherapy. We discovered that the ABCC2‐24C > T (rs717620) genotype was associated with the effectiveness of chemotherapy and patient outcomes.^6^ The relevance and mechanisms of gastric cancer need to be further identified.

ABCC2, a member of the ATP‐binding cassette subfamily C, functions as an efflux transporter, moving anionic drug conjugates such as glucuronates, sulfates, and glutathiones.^7^, ^8^ It also facilitates the transportation of anti‐cancer drugs, including cisplatin, vinblastine, and camptothecin derivatives.^9^ Several studies prove that overexpression of ABCC2 makes the tumour cell acquire multi‐drug resistance.^8^, ^10^ However, recent studies revealed that ABCC2 was associated with an increased disease‐free survival rate in colon cancer patients treated with FOLFOX‐4.^11^ Increased ABCC2 expression in colorectal cancer cells has been shown to enhance the response to oxaliplatin therapy.^12^ This finding suggests that ABCC2 may function as an anti‐cancer agent through alternative pathways in tumour progression, beyond its established role in multi‐drug resistance in gastric cancer.

As a substrate of ABCC2, glutathione (GSH) serves as the primary antioxidant in mammalian cells and is the preferred substrate of GPX4.^13^, ^14^ Depletion of GSH directly impacts GPX4 activity and stability, making cells more susceptible to ferroptosis,^15^, ^16^ a distinct form of regulated cell death characterized by extensive lipid peroxidation resulting from metabolic dysfunctions.^17^, ^18^ Numerous amino acid metabolic pathways have recently been identified as converging on ferroptosis. For example, glutaminolysis, which fuels the tricarboxylic acid (TCA) cycle, has been implicated in necrotic cell death triggered by amino acid starvation, ultimately leading to ferroptosis.^19^ While ABCC2 is essential for the efflux of GSH from hepatocytes into the bile canaliculus, its role in glutathione metabolism and ferroptosis in gastric cancer remains unclear.

In this study, we aim to explore the mechanism underlying the ABCC2‐24C > T (rs717620) genotype correlation with chemotherapy efficacy and prognosis, with the assumption that ABCC2 could induce metabolic vulnerability and ultimately ferroptosis through glutathione efflux.

## 2 MATERIALS AND METHODS

The detailed Methods have been provided in the Supporting Information Methods (Additional file 1).

### 2.1 Clinical specimen collection

Human gastric cancer tissues were obtained from 1044 GC patients undergoing radical resections, of which 238 GC patients received neoadjuvant chemotherapy at the Peking University Cancer Hospital between January 2005 and December 2010. Twenty‐two patients who developed distant metastases were excluded from the analysis. These samples were used for immunohistochemistry (IHC) staining, and 80 untreated patients were selected for droplet digital polymerase chain reaction (ddPCR) analyses. The TNM stage of GC was determined based on the 8th edition of the Cancer Staging Manual by AJCC. The TNM stage of gastric cancer was determined according to the 8th edition of the Cancer Staging Manual by the AJCC. Additionally, 74 gastric cancer and adjacent normal (paracarcinoma) tissue samples from patients underwent whole‐exome sequencing (WES) and RNA sequencing (RNA‐seq). WES and RNA‐Seq were performed as previously described.^20^ The details of WES and RNA‐seq can be found in the Supplementary Methods.

All the participants provided written informed consent, and the Ethics Committee of Peking University Cancer Hospital approved this study (approval number: 2019KT10). Overall survival (OS) was calculated from the surgery date to the date of death for any reason. The details of clinical characteristics are summarized in Table 1.

**TABLE 1:** Correlation between ABCC2 expression levels and clinicopathological features in patients with gastric cancer.

Variables	Patients	ABCC2 IHC(%)		*X* ^2^	*p‐*value
		−/+	++/+++		
Sex					
Male	782	675(86.3)	107(13.7)	2.355	0.125
Female	262	216(82.4)	46 (17.6)		
Age					
≤60	528	455(86.2)	73(13.8)	.588	0.443
>60	516	436(84.5)	80(15.5)		
pTNM^a^					
I	124	102(82.3)	22(17.7)	6.481	**0.039**
II	314	262(83.4)	52(16.6)		
III	548	486(88.7)	62(11.3)		
*N* ^a^					
0–1	466	383(82.2)	83(17.8)	7.405	**0.007**
2–3	567	500(88.2)	67(11.8)		
Vascular invasion					
Negative	510	433(84.9)	77(15.1)	1.192	0.275
Positive	503	439(87.3)	64(12.7)		
Differentiation					
Poor	451	409(90.7)	42(9.3)	17.563	**<0.001**
Moderately	507	412(81.3)	95(18.7)		
Well	27	22(81.5)	5(18.5)		
Lauren type					
Intestinal	602	498(82.7)	104(17.3)	12.935	**0.002**
Diffused	223	204(91.5)	19(8.5)		
Mixed	176	158(89.8)	18(10.2)		

*Note*: *p*‐values in boldface type indicate significance at a *p*‐value of <0.05.

^a^ All staging was based on the eighth edition of the AJCC Cancer Staging Manual by the American Joint Committee on Cancer and the International Union against cancer.

### 2.2 Immunohistochemistry

As previously described,^21^ after dewaxing and gradually hydrating, the tissue sections were heated at 95°C for antigen retrieval in pH 6.0 citric acid buffer (Sigma‐Aldrich). To block endogenous peroxidase and non‐specific binding sites, the sections were exposed to 3% H2O2 for 10 min. Next, the tissue samples were treated overnight at 4°C with the primary anti‐body targeting ABCC2 (dilution 1:1000, catalogue number ab998 from Abcam). This was followed by the application of the ImmPRESS Peroxidase Polymer Detection Reagent from Vector Laboratories, and the immunostaining was visualized using DAB. After counterstaining with Mayer's hematoxylin (Sigma‐Aldrich), the stained specimens were viewed under a light microscope (Nikon Corporation). The images were quantified based on the intensity of staining and the proportion of stained cells by two experienced pathologists blinded to the patient's clinical outcomes. The final IHC score was recorded as follows: −/+ (low expression of ABCC2), +/++ (high expression of ABCC2).

### 2.3 ddPCR and single nucleotide variant genotyping

The DNA was extracted from tissue samples by utilizing the DNeasy Blood & Tissue Kit (#69504, Qiagen). A panel of 23 single nucleotide variants (SNPs) linked to resistance against platinum and 5‐Fu‐based therapies was selected from the pharmGKB database (Table S1). Following this, ddPCR was utilized on the extracted DNA using the SNaPShot SNP multiplex genotyping assay (Life Technologies) in accordance with the provided instructions. The ABI 3130 Genetic Analyzer (Life Technologies) was utilized for capillary electrophoresis, followed by analysis of the results using GeneMapper software version 4.0 (Life Technologies). The sequence of the used primers and probes for ddPCR are listed in Supporting Information Methods.

### 2.4 Cell lines

Cell lines SGC7901, BGC823, and MGC803 derived from GC were acquired from the Cell Research Institute at the Chinese Academy of Sciences in Shanghai, and AGS cells and HEK293T cells were obtained from the American Type Culture Collection. The Supporting Information Methods section provides additional information on the cell culture specifics and the creation of ectopic expression or knockout cell lines.

### 2.5 Germline mutation calling

The details of WES sequencing and data preprocessing are available in the Supporting Information Methods section. To ensure the quality of FASTQ files, Trimmomatic version 0.36 was utilized for initial quality checks, securing only the high‐quality reads for further analysis. These processed reads were aligned against the reference human genome (hg19, GRCh37) using the Burrows‐Wheeler Aligner version 0.7.12. Duplicate removal was executed using Picard tools, followed by local realignment around insertions and deletions (indels) and base quality score recalibration, employing the Genome Analysis Toolkit (GATK version 3.2). Furthermore, single nucleotide polymorphisms (SNPs) in normal control samples were detected using GATK (version 4.2.3.0), focusing on SNPs with an allele frequency greater than 20% and observed in at least 1% of the population. Somatic single‐nucleotide variants (SNVs) and short insertions/deletions (indels) were identified utilizing VarDict. The final list of mutations was annotated using vcf2maf.

### 2.6 Luciferase reporter analysis

Wild‐type and SNP (rs717620 C > T) of ABCC2 3′‐UTR were synthesized and inserted into the p‐MIR‐REPORT plasmid to perform luciferase reporter experiments. Then, wild or SNP type of 3′‐UTR of ABCC2 accompanied with ETS1 or SOX9 were transfected into HEK293T cells using Lipofectamine 2000 reagent. Forty‐eight hours after transfection, the cells were harvested and lysed. Subsequently, the luciferase activity was analyzed with a Luciferase Assay Substrate (Promega).

### 2.7 Statistical analysis

All statistical analyses were performed utilizing GraphPad Prism 8.0 (GraphPad Software Inc.), and all data were expressed as mean values ± SD. Student's *t*‐test was employed to compare two groups, and multiple groups were compared using one‐way variance analysis. The chi‐square test was used to identify the association between ABCC2 expression levels and clinicopathological features of GC patients. The survival curves were drawn by Kaplan‐Meier analysis and the log‐rank test was used to compare the survival differences. Univariate and multi‐variate Cox proportional hazards model was utilized to evaluate the effects of clinical parameters on survival. Differences were defined as significant: *p < 0.05; **p < 0.01; ***p < 0.001.

## 3 RESULTS

### 3.1 The ABCC2‐24C > T variation correlates with increased expression levels and improved clinical results in individuals with gastric cancer

**FIGURE 1:**
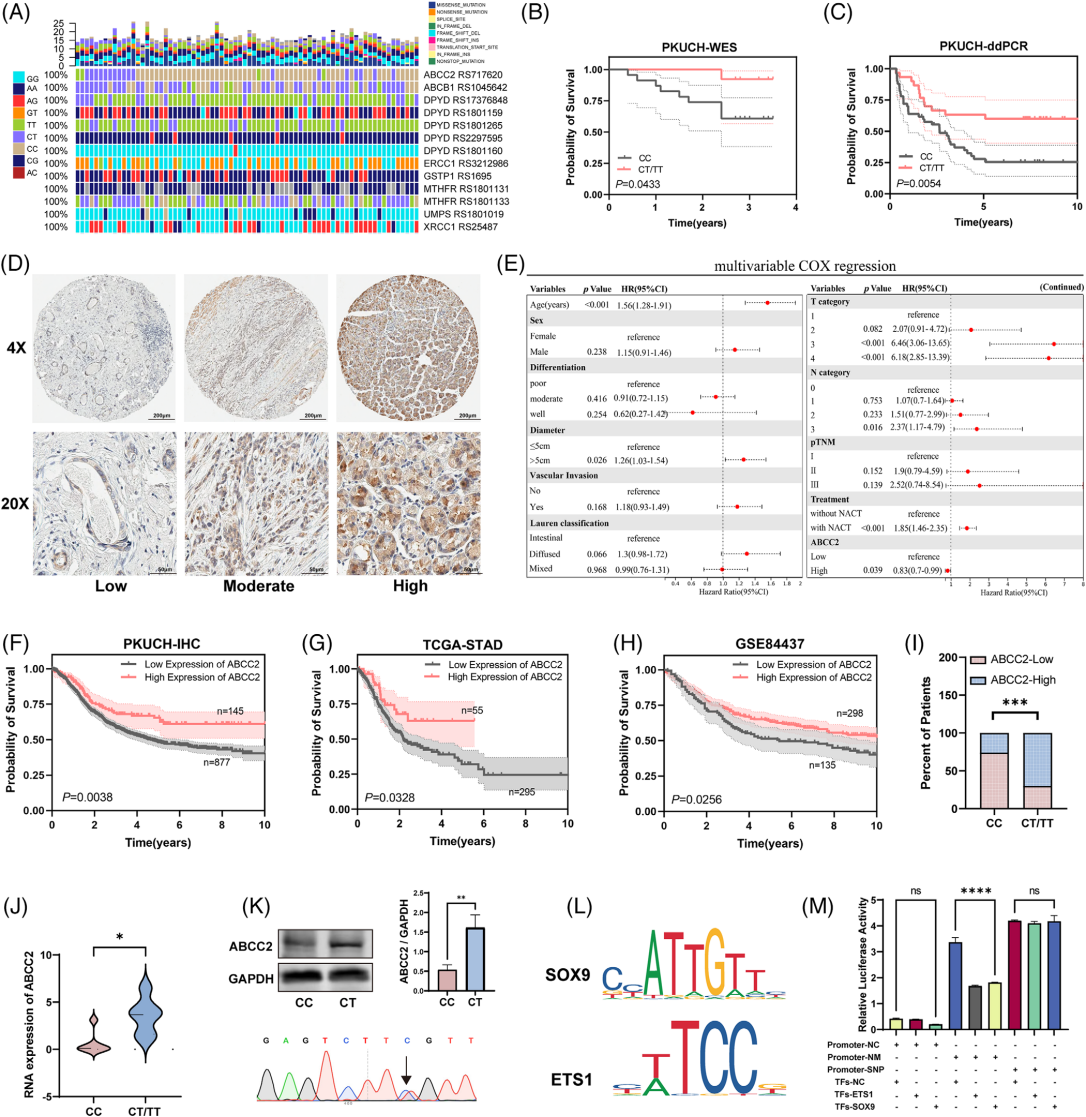
ABCC2‐24C > T polymorphism is associated with better clinical outcomes in gastric cancer patients. (A) The mutational landscape for the SNPs related to platinum/5‐Fu‐based chemotherapy resistance in this study. (B&C) Survival analysis of ABCC2 rs717620 and the wild type in cohort 1(left) and validation cohort (right). (D) Immunohistochemistry using DAB (brown), showing the expression of ABCC2 in gastric cancer tissues. Scale bar: 200 and 50 µm. Low (−/+), Moderate (++), High (+++). (E) Multi‐variate Cox regression analysis for assessment of the prognostic parameters of ABCC2 expression and different clinicopathological characteristics. (F) Kaplan‐Meier survival analysis of OS according to ABCC2 levels in 1022 GC patients for gastric cancer. (G, H) Kaplan‐Meier survival analysis of OS according to ABCC2 levels in the TCGA database and GSE84437 dataset. (I, J) The IHC (I) and mRNA (J) expression levels of ABCC2 in patients with the ABCC2‐24C > T genotype. *p < 0.05, ***p < 0.001; Student's *t*‐test. (K) CRISPR/Cas9 monoclonal screen to stably express the ABCC2 point mutation (24C > T) in the gastric cancer cell line SGC‐7901. The expression of ABCC2 was subsequently determined and quantified by western blot analysis. (L) Predicted transcription factors based on the ABCC2‐24C > T variant. (M) The dual luciferase assay showed SOX9 and ETS1 could significantly repress luciferase expression in the normal genotype. SGC‐7901 cells were cotransfected with either empty vector plasmid (Promoter‐NC), or the ABCC2 promoter plasmid (promoter‐NM/promoter‐SNP) and SOX9 or ETS1 expression plasmid.

To comprehend the genetic characteristics of gastric cancer, we performed WES and RNA sequencing on 74 samples of gastric cancer, using the adjacent normal tissue sample from the same patient as the control (Figure 1A; Figure S1A, B, Cohort 1). The mean sequencing coverages for WES were 224.1× and 117.8× for tumour tissues and matched normal samples, respectively. A total of 13 226 genetic changes were identified, consisting of SNVs and InDels, with a median of 96 alterations per individual (Figure S1C). Specifically, the ABCC2‐24C > T polymorphism was strongly associated with a favourable prognosis for gastric cancer (Figure 1B). To validate these findings, we performed ddPCR on 80 samples to detect the ABCC2‐24C > T polymorphism and found that patients with the ABCC2‐24C > T genotype had significantly better outcomes (Figure 1C).

The expression levels of ABCC2 were analyzed in 1044 gastric cancer samples from cohort 2, including 238 cases that received neoadjuvant therapy, using immunohistochemistry (Figure 1D). Comprehensive clinicopathological parameters of the patients are detailed in Table 1. ABCC2 expression was found to be significantly correlated with the earlier pTNM stage (*p *= 0.039), N stage (*p *= 0.007), lower differentiation degree (*p *< 0.001), and intestinal Lauren type (*P *= 0.002), thus conferring a favourable prognosis in patients with elevated ABCC2 expression (Table 1, Cohort 2). Additionally, univariate and multi‐variate Cox regression analysis identified ABCC2 expression level (HR: .83, 95% CI: .77–.99, *p* = 0.039) as a protective factor for GC prognosis. Meanwhile, age, pTNM stage, T stage, N stage, differentiation, Lauren classification, and vascular invasion were risk factors for GC prognosis (Figure 1E). Intriguingly, the Kaplan–Meier survival analysis showed that patients with gastric cancer who had increased ABCC2 expression had a significantly better overall survival rate (*p *= 0.0015 in 1044 total cases, Figure 1F). ABCC2 was associated with a better prognosis and validated in the TCGA and GSE84437 datasets (Figure 1G, H).

Furthermore, IHC and RNAseq results indicate a significant correlation between higher ABCC2 expression levels in patients and the ABCC2‐24C > T genotype (Figure 1I, J). A CRISPR monoclonal screen was employed to introduce the ABCC2 point mutation (rs717620) into the SGC‐7901 cell line, resulting in a notable rise in ABCC2 expression in the SNP cell line (Figure 1K). The ABCC2‐24C > T variant is located in the promoter region, and bioinformatics prediction suggested that it may impact ABCC2 transcription through binding with SOX9 and ETS1 (Figure 1L; Figure S1D). To explore the specific influence of ABCC2‐24C > T on ABCC2 transcription, a dual luciferase assay was performed, revealing that SOX9 and ETS1 could significantly repress the expression of luciferase in the normal genotype, but their ability to suppress was markedly reduced in the ABCC2‐24C > T genotype (Figure 1M).

### 3.2 Starvation causes a significant decrease in the migration and proliferation capacity of ABCC2 high‐expression cell lines

To investigate the possible role of ABCC2 in gastric cancer, we utilized our data (Figure S2A) and TCGA RNA‐seq (Figure S3A) expression profiles of gastric cancer to uncover the potential mechanisms of ABCC2 in GC. Using the RNA‐sequencing information from GC samples at PKUCH, we identified 8659 genes that were expressed differently, including 6996 genes that were upregulated and 1663 genes that were downregulated (Figure S3A). Analyzing the functional enrichment of the genes with differential expression provided insight into their biological significance. In the KEGG pathway and GO term enrichment analysis, several enriched pathway terms were enriched in the oxidative phosphorylation, amino acid, and glutathione metabolic pathway (Figure S3B, C). GSEA analyses showed the amino acid metabolic pathway and oxidative stress gene set to be positively enriched following ABCC2 increased expression (Figure S3D, E).

Using RNA‐sequencing data from 386 GC samples in TCGA, we discovered 429 genes expressed differently, including 401 genes that were upregulated and 28 genes that were downregulated (Figure S3F). Enrichment studies also indicated that the genes with altered expression were mainly associated with a variety of metabolic pathways, such as fat digestion and absorption, carbohydrate digestion and absorption, central carbon metabolism in cancer, protein digestion and absorption, and protein–lipid complex assembly. ABCC2 expression showed a significant correlation with reactive oxygen species (ROS) levels and various amino acid metabolic pathways, such as glutathione metabolism and glycine, serine, and threonine metabolism. This indicates the important role of ABCC2 in controlling amino acid metabolism and oxidative stress in gastric cancer cells (Figure S3I). Interestingly, we performed GSEA and KEGG enrichment analyses on differential expression genes from patient samples and the isogenic cell lines with the rs717620 variant or wild‐type samples and identified significant enrichment of pathways related to amino acid metabolism, oxidative stress, and ferroptosis (Figure S2A–F). These suggest that the impact mechanism of the ABCC2 rs717620 variant, situated on the promoter region, on tumours, resembles the influence of ABCC2 expression on tumorigenesis.

**FIGURE 2:**
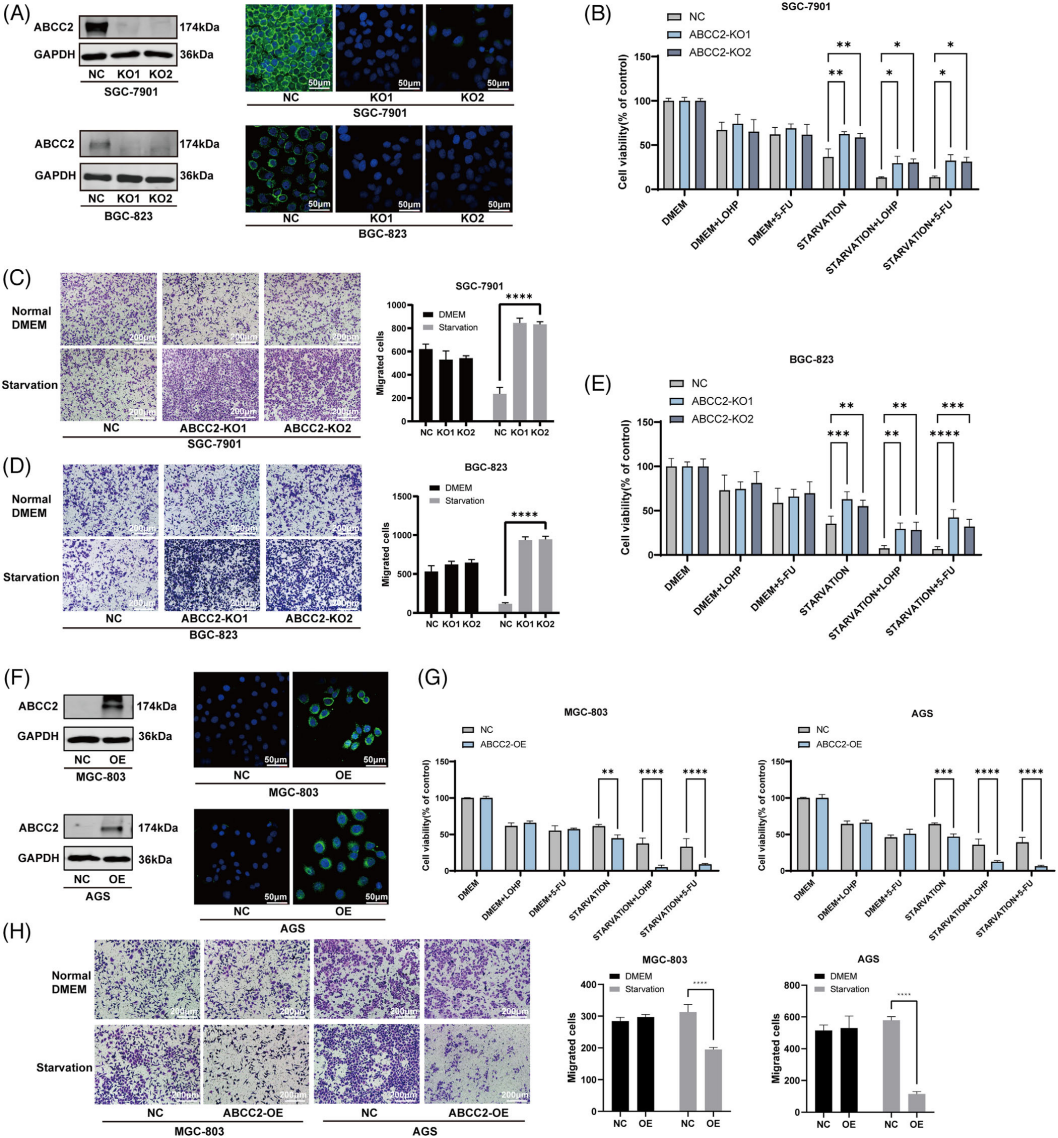
Starvation impairs the migration and proliferation capacity of ABCC2 high‐expression cell lines. (A) The knockout efficiency of ABCC2 was determined by western blot and immunofluorescence in BGC‐823 and SGC7‐901 cells. Green: ABCC2 immunofluorescence; blue: DAPI. (B–E) Proliferation and migration assays were performed to examine the biological function in ABCC2‐ knockout BGC‐823 and SGC‐7901 cells. (F) The efficiency of ABCC2 overexpression was determined by western blot and immunofluorescence in MGC‐803 and AGS cells. Green: ABCC2 immunofluorescence; blue: DAPI. (G, H) Proliferation and migration assays were performed to examine the biological function in ABCC2 overexpressed MGC803 and AGS cells. *p < 0.05, **p < 0.01, ***p < 0.001 vs. the vehicle control (NC group); Student's *t*‐test.

To investigate the potential effects of ABCC2 on GC cell growth and migration under different conditions, we established stable ABCC2 knocked‐out cell lines (BGC823 and SGC7901) and ABCC2 overexpressed cell lines (MGC803 and AGS) (Figure 2A, F). Under starvation conditions, ABCC2 knockout enhanced the proliferation and migration of SGC7901 and BGC823 cells (Figure 2B–E; Figures S4B and S5B), while overexpression of ABCC2 significantly inhibited the proliferation and migration of MGC803 and AGS cells (Figure 2G–H; Figures S4A and S5A). Notably, cells with higher ABCC2 expression showed markedly higher sensitivity to 5‐FU and LOHP under starvation conditions. However, following the amino acid supplementation, the expression of ABCC2 does not influence cell proliferation, migration, and 5‐FU and LOHP resistance. These findings suggest that ABCC2 plays a crucial role in regulating cell growth and migration under starvation conditions and that its expression level affects the sensitivity of GC cells to 5‐FU and LOHP.

### 3.3 Oscillation of ABCC2 expression can entrain fluxes in cellular amino acid and energy production by reprogramming cellular metabolism

**FIGURE 3:**
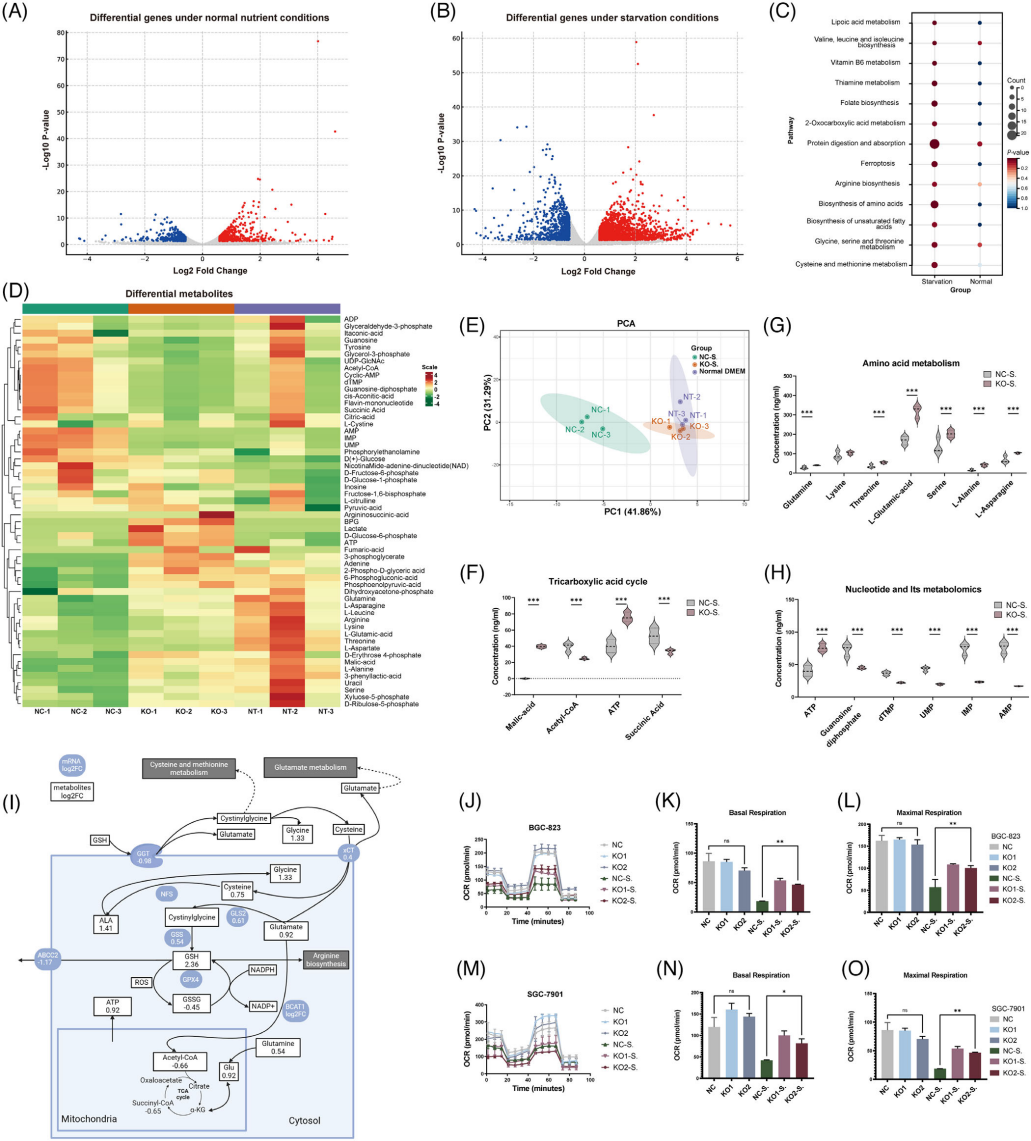
ABCC2 induces metabolic vulnerability and impairs cellular OXPHOS under starvation conditions. (A, B) Volcano plots showing differentially expressed genes between ABCC2‐KO and control SGC‐7901 cells with (A) or without (B) starvation. ABCC2 knockout cells displayed more differentially expressed genes under starvation conditions. *P*‐value derived from statistical testing, typically a *t*‐test. Genes that significantly increased or decreased were coloured in red and blue, respectively. (C) KEGG pathway enrichment results of differential gene expression in ABCC2‐knockout and control cell lines under different culture conditions. (D) A hierarchical clustering heatmap was generated to visualize the patterns of central carbon metabolites detected using the LC‐ESI‐MS/MS system. The samples included Control (NC‐S.) and ABCC2‐knockout (KO‐S.) SGC‐7901 cells were cultured in the amino acid‐free medium for 12 h. (E) Principal component analysis (PCA) was performed to visualize the patterns of central carbon metabolites detected using the LC‐ESI‐MS/MS system. Each point in the graph represents a sample, and samples from the same group are represented with the same colour. The vehicle control with amino acid‐free medium (NC‐S group), the ABCC2‐KO group with amino acid‐free medium (KO‐S group), and normal DMEM (NT group). (F–H) Differential metabolites involved in glycolysis, the pentose phosphate pathway (PPP), the tricarboxylic acid (TCA) cycle, and amino acid metabolism using an LC‐ESI‐MS/MS system in control SGC 7901 cells versus ABCC‐KO cells. ***VIP (variable importance in projection) >1. (I) Differential central carbon metabolism‐related metabolites and gene expression in ABCC2‐knockout and control cell lines. Blue ellipses represent mRNAs, and black squares represent metabolites. (J–O) Oxygen consumption rate (OCR) measured in ABCC2‐KO or its control SGC7901 and BGC 823 cells using the Seahorse XF Cell Mito Stress Test.

We next conducted RNA‐Seq analysis on ABCC2 knockout and control cell lines under various culture conditions. The comparison between the ABCC2 knockout (KO) and control SGC‐7901 cell lines (NC) cells under normal nutrient conditions showed 419 upregulated and 405 downregulated differentially expressed genes (Figure 3A). Notably, under starvation conditions, ABCC2 knockout cells displayed more differentially expressed genes, with 2389 genes upregulated and 903 genes downregulated (Figure 3B). This observation suggests that ABCC2 involves a broader range of mechanisms in starvation environments, as illustrated in Figure 3I. The increased expression of a substantial set of differential genes associated with metabolism and metabolism regulation was observed in ABCC2 knockout cells under starvation conditions, as depicted by the top enriched KEGG terms (Figure 3C). Moreover, integrated analysis of RNA data from TCGA patients and our RNA data from PKU patients and cells showed significant enrichment in pathways related to amino acid metabolism and oxidative stress in both enrichment analysis and GSEA.

To elucidate the impact of ABCC2 on GC metabolism, cells from the ABCC2 knockout (KO group) and vehicle SGC‐7901 cells (NC group) were grown in an amino acid‐deprived medium or regular DMEM for 12 h. Subsequently, metabolites were extracted for targeted metabolomics analysis of key carbon metabolites related to glycolysis, the pentose phosphate pathway (PPP), the tricarboxylic acid (TCA) cycle, and amino acid metabolism utilizing an LC‐ESI‐MS/MS system. This analysis aimed to assess the metabolic alterations induced by ABCC2 in these specific pathways. The heatmap and principal component analysis demonstrated that the energy metabolism patterns in ABCC2 knockout cells treated with amino acid‐free medium were similar to those of the control cells cultured in normal DMEM (NT group). However, distinct metabolic patterns were observed between the NC and KO groups with amino acid‐free medium (Figures 3D, E; Figure S6A). The findings from the analysis indicate that ABCC2 plays a significant role in modulating energy metabolism and leads to notable metabolic alterations, especially when comparing the NC group with the KO group in an amino acid‐free medium. The loss of ABCC2 led to a significant increase in ATP and the several carbon sources for the tricarboxylic acid (TCA) cycle (glutamine increased to 145.8% and Asparagine increased to 150.3%), and moderate accumulation of metabolic intermediates in the TCA cycle, such Acetyl‐CoA (by 147.8%) (Figure 3F), malic‐acid and succinic acid (by 56.2%) and almost all amino acid metabolites (Figure 3G). Significant decreases were also observed with internal dTMP, UMP, IMP, and AMP in ABCC2‐KO cells (Figure 3H; Figure S6B–D). These findings imply that ABCC2 impairs mitochondrial respiration and TCA cycle activity in an amino acid‐free medium (Figure 3I; Figure S7A, B).

To confirm the metabolic changes in ABCC2 knockout cells, we analyzed their glycolytic activity through ECAR measurement and their oxidative phosphorylation rate through OCR measurement using the Agilent Seahorse XF analyzer. The measurement of OCR demonstrated that mitochondrial respiration was diminished in the control cells compared with ABCC2‐KO SGC‐7901 or BGC‐823 cells, as evidenced by a 38.3% reduction in basal respiration, a 53.1% reduction in the maximal respiratory capacity, and a 47.6% reduction in OCR‐coupled ATP production (Figure 3J–L). Under starving conditions, the ABCC2 knockout cells demonstrated higher basal OCR relative to control cells with elevated maximum glycolytic capacity and mitochondrial respiration, suggesting ABCC2 promotes cellular metabolic reprogramming by disturbing acid metabolic pathways (Figure 3J–O).

### 3.4 Starvation leads to GSH metabolic reprogramming and more sensitivity to oxidative stress in ABCC2 high cells

**FIGURE 4:**
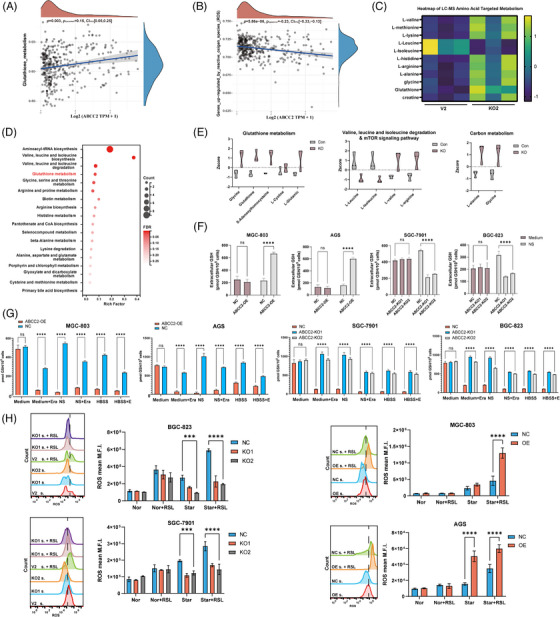
ABCC2 causes a significant decrease in cellular amino acid levels by reprogramming cellular metabolism. (A, B) The correlations between ABCC2 and the different pathway was analyzed with Spearman. (C) Heatmap plots of metabolite alterations in ABCC2‐KO SGC7901 cells or its V2 control. (D) KEGG metabolic pathways enriched by significantly differential metabolites in the control group (V2) versus ABCC‐KO SGC‐7901 cells. One‐sided Fisher's exact test followed by BH multiple comparison test with FDR < .05 and VIP > 0. (E) Violin plots of differential metabolites in control SGC 7901 cells versus ABCC‐KO cells. (F–G) Intracellular and extracellular glutathione concentrations detected by glutathione assay kit in gastric cancer cell lines. (H) Detection of endogenous ROS in gastric cancer cell lines treated with RSL3 under different nutrient conditions.

Regulation of intracellular glutathione levels is essential for maintaining appropriate levels of ROS and the ferroptosis process. ABCC2, a plasma membrane protein, has been identified to mediate GSH extrusion. Correlation analysis of TCGA data reveals a correlation between glutathione metabolism, regulation of ROS, and ABCC2 (Figure 4A, B). Due to GSH's significant impact on the amino acid pool, we conducted targeted metabolomics using selective ion mode GC‐MS to quantify a wide range of amino acids, such as GSH, GSSG, glycine, cysteine, glutamate, glutamine, and acetylated amino acids. The heatmap generated from GC‐MS data, depicted in Figure 4C, revealed a significant shift in metabolite concentrations in ABCC KO compared with the control group. Total amino acid levels were significantly elevated in the ABCC2 knockout cell line compared to control under starvation conditions, and glutathione was among the highest metabolic substrates, while oxidized glutathione was unchanged, resulting in an increased GSH/GSSG ratio (Figure 4E). Furthermore, KEGG pathway‐based analysis showed several amino acids metabolic pathways, such as Glutathione metabolism, valine, leucine, and isoleucine biosynthesis, and glycine, serine, and threonine metabolism, was disturbed in ABCC2 high cell lines (Figure 4D).

To unravel the causes of GSH decrease in the absence of GSSG increase, we evaluated whether this phenomenon was due to an augmented efflux of the tripeptide toward the extracellular space by measuring the presence of GSH in the cell medium. A GSH and GSSG assay kit was employed to measure intracellular/extracellular GSH and GSSG levels after 12 h of starvation. Our results demonstrate that ABCC2 leads to a significant increase in extracellular GSH concentration and a substantial decrease in intracellular GSH concentration after only 12 h of nutrient removal (Figure 4F, G). Under normal DMEM conditions, the intracellular GSH and GSSG levels remained unaltered, indicating that cells may rely on additional exogenous pathways for GSH replenishment. Notably, ABCC2 knockout cells exhibited a significant blockage of GSH efflux, leading to stable GSH/GSSG ratio (Figure S8A, B) and ROS levels (Figure 4H). These findings suggest that ABCC2 is critical in regulating intracellular GSH levels and ROS homeostasis under nutrient‐deprived conditions.

To further investigate whether the role of ABCC2 in GSH efflux is cell‐line‐specific, we established stable overexpression of ABCC2 in AGS and 803 cells. Figure 4F, G showed that ABCC2 overexpression led to a decrease in intracellular glutathione levels under starvation conditions, which was associated with the generation of ROS. These findings suggest that ABCC2 is crucial in mediating intracellular glutathione reduction and contributes to an altered oxidative stress state in gastric cancer cells.

### 3.5 Reduced glutathione levels due to starvation induce ferroptosis

**FIGURE 5:**
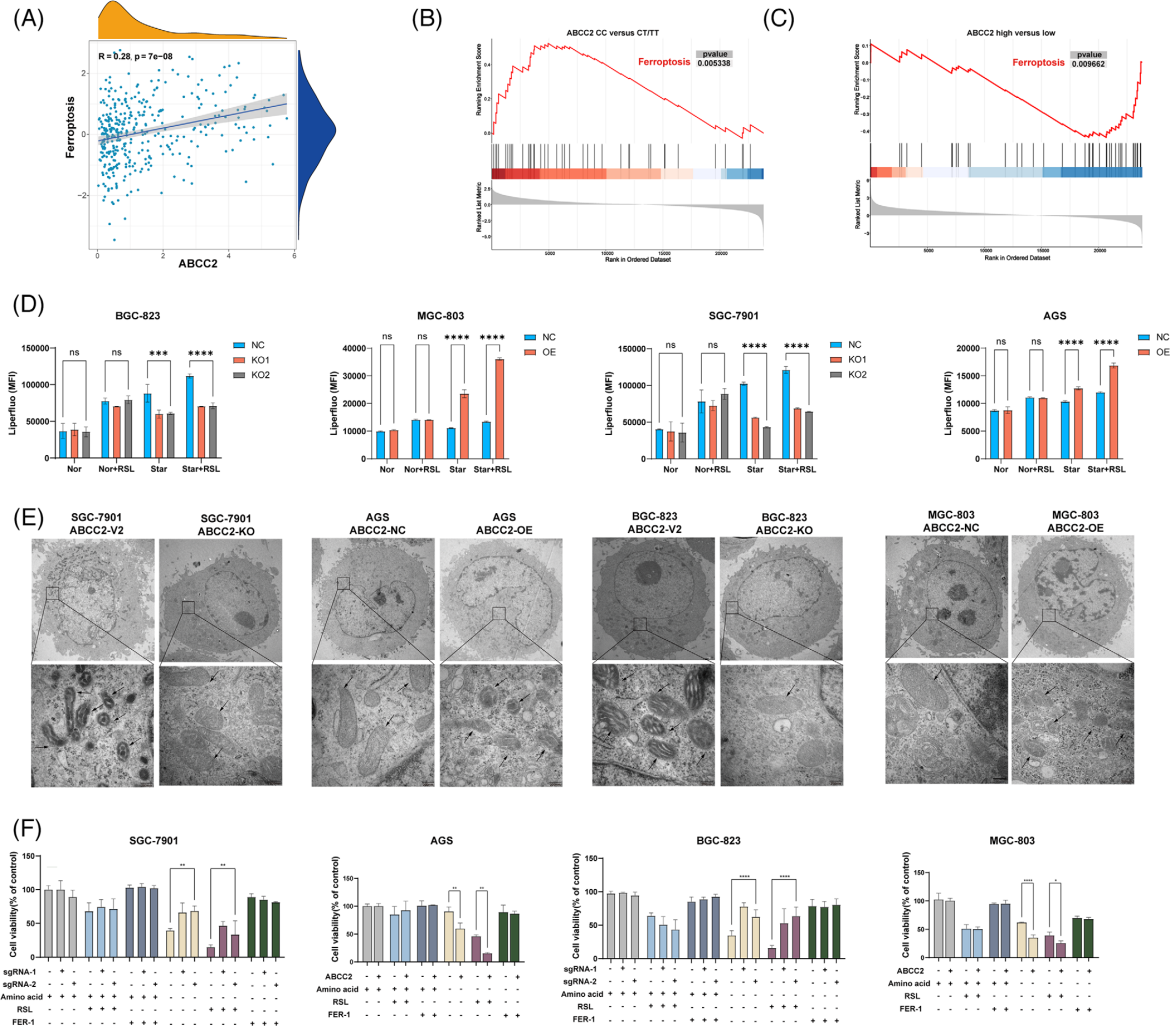
Liperfluo detection of intracellular lipid peroxidation changes under nutrient deprivation conditions simulating tumour microenvironment conditions. (A) The correlations between ABCC2 and the ferroptosis pathway were analyzed with Spearman in the TCGA‐STAD cohort. (B) GSEA analysis of differential gene expression between ABCC2‐CC and CT/TT genotype patients in PKUCH cohort. (C) GSEA analysis of differential gene expression between ABCC2‐high and low patients in PKUCH cohort. (D) Effects of starvation medium and RSL3 on LPO level of gastric cancer cell lines in a different expression of ABCC2, using Liperfluo as a probe for flow cytometry. (Nor: normal medium, S: Starvation medium, RSL: cultured with 10 µM RSL3 for 12 h). The error bars represent the standard error from three replicates. (E) Low‐ and high‐magnification transmission electron microscopy (TEM) images of cancer cells cultured with starvation conditions. Five image fields were examined from two independent experiments with consistent observations for each condition. (F) Proliferation assays were performed to examine the biological function in ABCC2 overexpressed MGC803 and AGS cells and ABCC2‐KO BGC823 and SGC7901 cells. *p < 0.05, **p < 0.01, ***p < 0.001 vs. the control group.

Correlation analysis of TCGA data reveals a correlation between ferroptosis gene features and ABCC2 (Figure 5A; Figure S9A). Furthermore, RNA sequencing results from both ABCC2‐knockout cell lines and tissue samples with low ABCC2 expression significantly enrich the ferroptosis pathway (Figure 5B, C; Figure S10A, B). The onset of ferroptosis occurs due to the disruption of regulatory redox processes, leading to extensive peroxidation of polyunsaturated phospholipids. We measured lipid peroxide (LPO) using liperfluo in different cell lines. Accumulation of LPO was observed in high expression levels of ABCC2 cell lines but not in the low expression level cells (Figure 5D). Transmission electron microscopy confirmed the occurrence of ferroptotic cell death. Incubation of high‐expressing ABCC2 cells under starvation conditions for 24 h increased mitochondrial membrane density and smaller mitochondria (Figure 5E), confirming that ABCC2 promotes nutrition deprivation‐induced ferroptosis. Meanwhile, we conducted western blotting to investigate the correlation between ABCC2 expression and SLC7A11 and GPX4 in gastric cancer cell lines under different nutritional conditions. As depicted in Figure S11, under normal nutritional conditions, ABCC2 expression did not significantly impact the levels of SLC7A11 and GPX4. However, under amino acid deprivation, heightened ABCC2 expression led to a significant decrease in GPX4 levels but did not affect SLC7A11 expression. Cell lines with varying levels of ABCC2 expression were exposed to the ferroptosis inducer RSL3 and inhibitors (ferrostatin‐1) to evaluate their susceptibility to ferroptosis. Compared with high‐expressing ABCC2 cell lines, cellular lipid peroxides were decreased in low‐expressing cells in response to RSL (*p* < 0.05) (Figure 5F). Ferrostatin‐1 greatly enhanced the survival of ABCC2‐overexpressing cell lines, suggesting that ferroptosis plays a role in their demise. The results indicate that the ABCC2 expression level influences the susceptibility of GC cells to ferroptosis.

### 3.6 ABCC2 synergizing with RSL3 to induce ferroptosis in vivo

**FIGURE 6:**
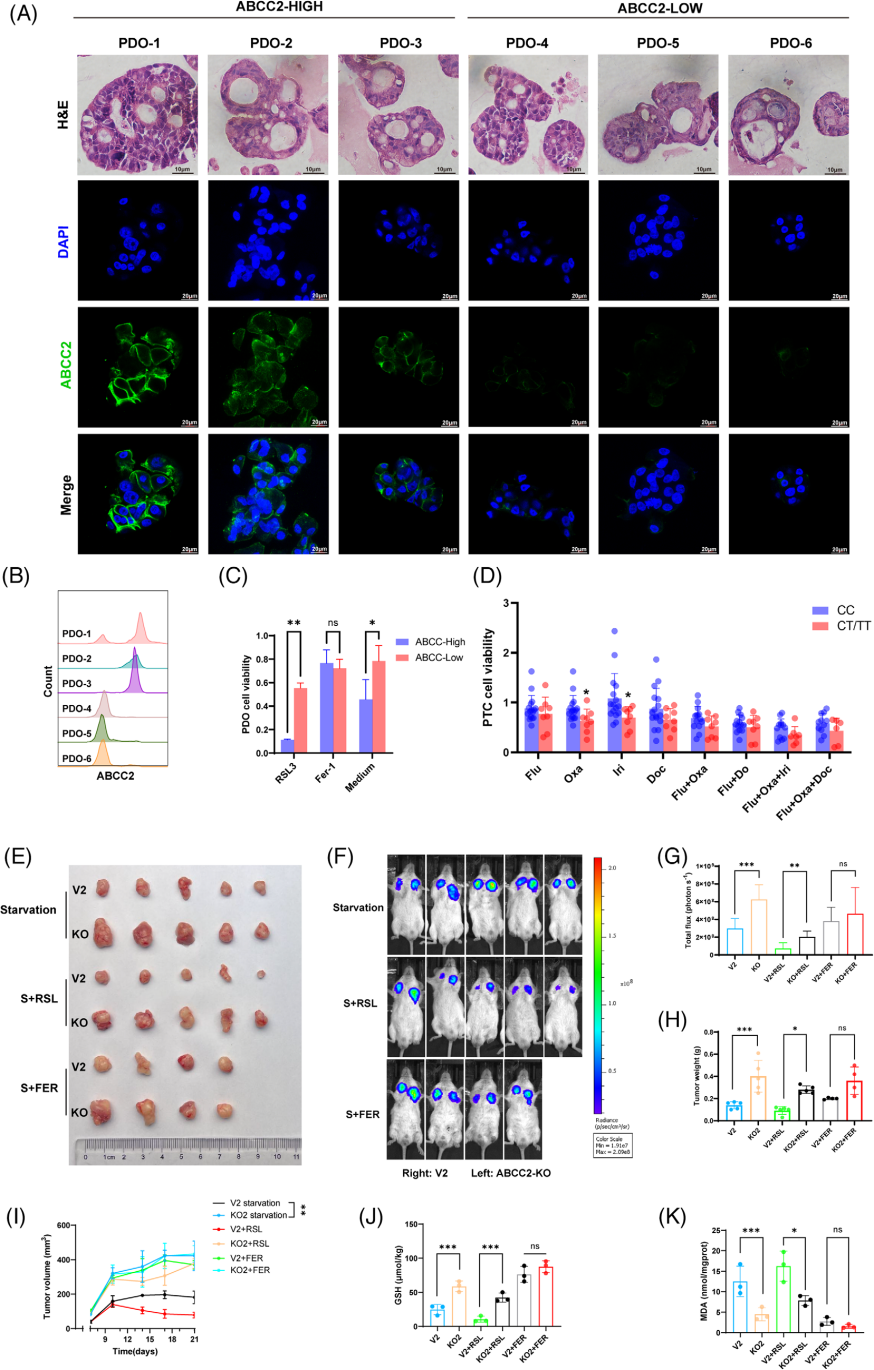
ABCC knockout gastric cancer cell line was significantly sensitive to ferroptosis inducers in vivo. (A) H&E and immunofluorescence staining of ABCC2 on serial sections of PDOs from six GC patients. Scale bars indicate 10 µm. (B) ABCC2 expression in PDOs was identified using flow cytometry. (C) Proliferation assays were performed to examine the cell viability of PDOs with ferroptosis triggers or inhibitors. (D) Proliferation assays were performed to examine the cell viability of PTCs with chemotherapy drugs. (E) The representative images and the quantification of xenograft in SGC‐7901 cells upon control (V2 plasmid) or ABCC2‐KO (cultured for 12 h in starvation medium) were implanted subcutaneously in NOD‐SCID mice (*n* = 5). (F) Representative images of mice and luciferase signal intensities are shown. (G) Luciferase signal intensities and tumour weights are represented as mean ± SD. (H) Tumour weights are represented as mean ± SD. (I) Tumour volumes were calculated after injection every 3 for 21 days. (J) Glutathione concentrations were detected by glutathione assay kit in xenograft of SGC‐7901 cells upon control (V2 plasmid) or ABCC2‐KO were implanted subcutaneously in NOD‐SCID mice. (K) MDA levels were evaluated in xenograft of SGC‐7901 cells upon control (V2 plasmid) or ABCC2‐KO were implanted subcutaneously in NOD‐SCID mice. *p < 0.05, **p < 0.01, ***p < 0.001 vs. the control group.

To further validate the role of ABCC2 in human gastric cancer, we established patient‐derived organoid (PDO) models using gastric cancer tumour tissues. We treated these organoids with RSL3 and assessed their growth (Figure 6A). The growth rates of the PDOs indicated that RSL3 more effectively inhibited the viability of organoids with low ABCC2 expression compared to organoids with high ABCC2 expression (Figure 6B, C). Additionally, Fer‐1 effectively enhanced the viability of organoids with high ABCC2 expression. Meanwhile, we used patient‐derived tumour‐like cell clusters (PTCs) to model the clinical relevance of ABCC2 24C > T. PTCs, collected immediately post‐resection, better mimic the tumour microenvironment and preserve the original tumour's genotype and phenotype, providing accurate predictions for chemotherapy outcomes.^22^ We observed that PTCs with ABCC2 24C > T exhibited significantly reduced resistance to oxaliplatin and irinotecan (Figure 6D). Similar trends were observed with other chemotherapeutic drugs.

Based on the aforementioned in vitro study results, we established a gastric xenograft mouse model by subcutaneously injecting NOD‐SCID mice with 7901 vector cells and 7901 ABCC‐KO cells. The tumour weight and size of the 7901 ABCC‐KO group were not significantly different from those of the control group under normal conditions (Figure S12A). In addition, our findings indicated that the SGC‐7901 ABCC‐KO group exhibited notably increased tumour weights and sizes compared with the control group when subjected to starvation conditions (Figure 6E). Significant decreases in tumour sizes and masses were noted in the high ABCC2 expression groups treated with RSL3 compared to the ABCC2 knockout group (*p* < 0.001; Figure 6F–I). Moreover, GSH levels significantly decreased in the control group with high ABCC2 expression, while MDA levels correspondingly increased significantly (Figure 6J, K), supporting the crucial role of ABCC2 in ferroptosis of gastric cancer cells. These findings further confirm the tumour‐suppressive activity of ABCC2 in GC and suggest its potential as a predictive biomarker for this disease.

## 4 DISCUSSION

A growing amount of genetic variations associated with gastric cancer risk have been identified as potential biomarkers for susceptibility to gastric cancer.^23^ Nevertheless, the comprehensive elucidation of whether these risk SNPs are linked to outcomes in gastric cancer has not been fully explored. In this research, a discovery is revealed that rs717620, located at 10q24, correlates with the effectiveness of chemotherapy and the outlook for GC patients as a hereditary alteration. Consistent with our results, multiple prior research studies have shown a connection between the ABCC2−24C > T mutation and the increased effectiveness of irinotecan^24^ or platinum‐based treatments.^25^ However, the underlying mechanisms of how this variant affects clinical outcomes are unclear. The rs717620 is positioned at 10q24 in the 5′UTR region of the ABCC2 transcript. Our joint examination of RNA sequencing and immunohistochemistry revealed that the ABCC2‐24C > T variation may lead to heightened ABCC2 levels. Early research suggests that these SNPs are likely to cause pathogenic effects by altering the binding of transcription factors to DNA via the creation or disruption of transcription factor binding sites, thereby leading to changes in downstream gene expression levels.^26^ We determined that transcriptional factors SOX9 and ETS1 act as negative regulators of ABCC2 expression—a mechanism that the rs717620 mutation can counteract. ABCC2 was shown to be a protective factor for patient prognosis based on 1048 immunohistochemical samples and TCGA data.

Moreover, the results discussed in this study offer new explanations for certain puzzling discoveries in cancer research. Previously, ABCC2 was reported as an anion pump protein highly expressed in various tumours and promotes tumour drug resistance through the efflux of chemotherapeutic agents.^27^ However, several articles have reported that high expression of ABCC2 is a marker of better prognosis in several cancer types,^11^, ^12^, ^25^ which is counterintuitive because one would assume that ABCC2‐mediated drug efflux would be beneficial to tumour cell growth. Indeed, we observed that overexpression of ABCC2 did not induce cell death, and there were no significant changes in IC50 to 5‐FU and oxaliplatin under normal culture conditions. So, what is the molecular basis underlying the tumour suppressive activity of ABCC2? By co‐analyzing RNAseq results from our own and TCGA databases, we identified consistent enriched pathways related to amino acid metabolism and oxidative stress.

Several reports have confirmed the deficiency of amino acids in the tumour microenvironment, and amino acid deprivation experiments can better simulate the conditions in the tumour microenvironment.^19^, ^28^, ^29^ Furthermore, the knockdown of ABCC2 in an amino acid deprivation environment led to a significantly higher number of differentially expressed genes (5027 genes) compared with the control medium (420 genes). The spectrum of altered genes suggests that metabolic stresses may influence the biological effects of ABCC2 on gastric cancer cells. Meanwhile, the viability of cells overexpressing ABCC2 was significantly reduced in an amino acid deprivation environment, and they exhibited increased sensitivity to fluorouracil and oxaliplatin. Through a series of gain‐ and loss‐of‐function experiments combined with metabolomics analyses, we identified ABCC2, a key molecule involved in GSH extrusion, as a critical factor in drug resistance.

To hypothesize the functional role of GSH efflux under starvation conditions, it should be considered that GSH is a highly abundant cellular tripeptide that can serve as a reservoir for the generation of new amino acid components. In our study, we found that additional pathways for GSH replenishment are crucial in gastric cancer cells. GSH synthesis involves two ATP‐dependent reactions catalyzed by glutamate‐cysteine ligase and glutathione synthetase (GSS).^30^ Cysteine is imported via the cystine‐glutamate reverse transporter, while glycine uptake and metabolism also contribute to GSH production.^31^ Glutamine, a major glutamate source, affects GSH levels through the regulation of glutaminase (GLS) activity and via the ASCT2 transporter.^32^ Glycine, besides its role in GSH synthesis, supports purine biosynthesis in cancer cells. Restricting serine and glycine levels reduces GSH synthesis and increases ROS.^33^ Interestingly, glycine metabolism promotes tumorigenesis, suggesting it is a potential therapeutic target. Analysis of over 200 metabolites in NCI‐60 cell lines revealed glycine consumption correlates with cancer cell proliferation rates, suggesting reliance on GSH for growth and survival. Indeed, disturbances in amino acid or glutathione levels in cancer have become attractive targets for combination therapy. Sulfasalazine and erastin are two of the most commonly used inhibitors of the glutamate transporter SLC7A11, and they can be used as therapeutic agents to enhance sensitivity to other drugs.^34^

Furthermore, we observed a significant regulation of genes encoding rate‐limiting enzymes, GSS, and GLS2,^35^ in the glutathione metabolism pathway after ABCC2 knockout. Crucially, ABCC2 overexpression results in a significant reduction in intracellular amino acid levels and diminished oxidative phosphorylation. Amino acid deficiency decreases mitochondrial membrane potential and leads to the early onset of cellular mitochondrial respiratory quiescence. A lack of amino acids reduces the potential of the mitochondrial membrane, causing cellular mitochondrial respiratory quiescence to start early. Metabolic reprogramming in ABCC2 high GC cells is characterized by the depletion of GSH for the production of essential cellular components. This leads to a reduced ability to clear ROS and a new vulnerability dependent on amino acids.

Intracellular GSH depletion is a crucial driver of ferroptosis induction. The GSH co‐enzyme allows GPX4 to remove lipid peroxides from cells according to reference.^28^ T The creation of GSH from its building blocks requires two enzymatic steps that use ATP: first, the production of γ‐glutamylcysteine from glutamate and cysteine, and second, the production of GSH from γ‐glutamylcysteine and glycine.^36^ GC cells exhibit reduced levels of GPX4 expression, rendering them more vulnerable to ferroptosis than healthy intestinal cells.^37^ Alborzinia et al.^29^ pharmacological blocking of cystine transport, transsulfuration, and GPX4 inactivation led to tumour regression in a MYCN‐amplified neuroblastoma model. Our results suggest that gastric cancer cells with high ABCC2 expression can affect the regulatory function of the cellular thiol pool and redox status by promoting GSH efflux in a starved environment, leading to increased cellular susceptibility to ferroptosis, a non‐apoptotic form of cell death. Additionally, in the gastric xenograft mouse model characterized by high ABCC2 expression, we noted that restricting amino acid intake in combination with GPX4 inactivation led to significant tumour regression. This finding underscores the potential of a multi‐pronged approach targeting ferroptosis as a promising therapeutic strategy for GC patients with high ABCC2 expression or ABCC2‐24C > T.

Yet there are some inadequacies in this study. We primarily enrolled GC patients from the Han Chinese population in northern China (Figure S13A, B). Therefore, it is essential to validate the relationship between rs717620 and clinical benefits in other populations with different genotypes. Additionally, the extensive amino acid starvation used to mimic metabolic stress in the tumour microenvironment suggests that ABCC2 could serve as a marker for overcoming GC resistance and is closely associated with amino acid metabolism. Further confirmation is needed to determine which specific amino acids of ABCC have the most significant effect on ABCC2. Moreover, the impact of ABCC2 on tumour prognosis may extend beyond cancer cells themselves, potentially enhancing the anti‐tumour ability of immune cells through glutathione efflux. The alteration of the tumour microenvironment resulting from glutathione efflux by ABCC2 is also worthy of further observation in subsequent studies.

**FIGURE 7:**
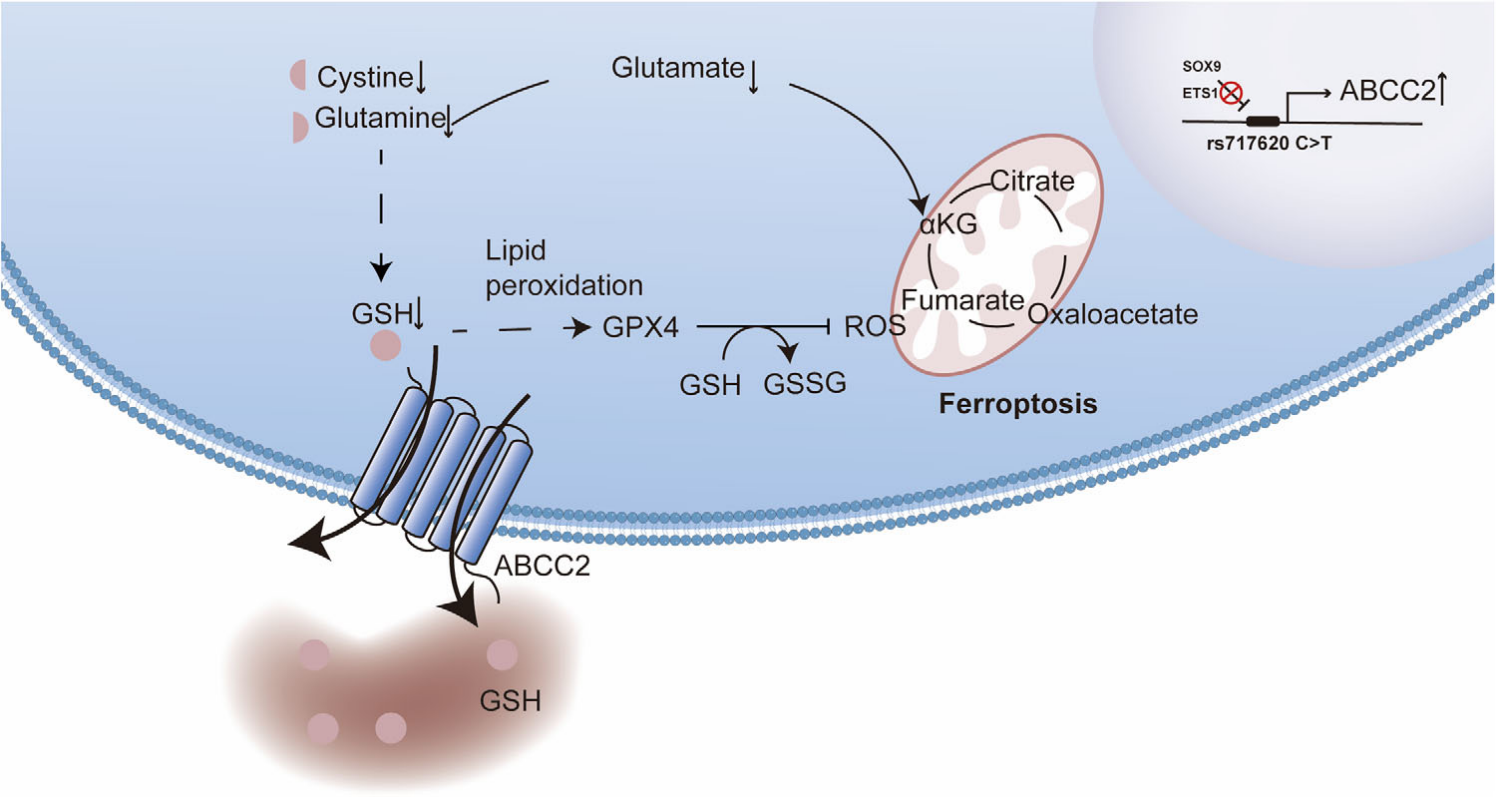
Schematic diagram representation of the mechanism of ABCC2 promoting ferroptosis in GC.

In conclusion, the present study has explored and preliminarily elucidated, for the first time, the potential mechanisms by which genetic polymorphisms contribute to diverse treatment responses and survival in different gastric cancer patients. The ABCC2‐24C > T polymorphism, located at 10q24 (rs717620), was associated with chemotherapy efficacy and prognosis. ABCC2‐24C > T increased ABCC2 expression through transcriptional regulation, leading to high ABCC2 expression inducing intracellular amino acid metabolism disorders via glutathione efflux. This process also resulted in increased oxidative stress and induced ferroptosis in tumour cells (Figure 7). Consequently, tumour cell proliferation and invasion are decreased, and chemotherapy sensitivity is increased, ultimately resulting in a better prognosis. Additionally, the study suggests that enhancing chemotherapeutic response by combining ferroptosis and amino acid uptake targets may be a potential therapeutic strategy for individuals with high ABCC2 expression.

## 5 CONCLUSION

In this study, we conducted a comprehensive analysis based on multi‐cohorts to elucidate that ABCC2 can induce AA metabolism disruption through glutathione efflux, mediating the induction of lipid peroxidation, ultimately leading to ferroptosis in GC cells. Our research highlights the potential to merge various ferroptosis targets as a treatment plan for GC with elevated ABCC2 levels, which have the potential to shed light on the pathogenesis of GC and contribute to the development of novel treatment strategies.

## AUTHOR CONTRIBUTIONS

Yiding Wang was involved with the study concept and design, acquisition of data, and drafting of the manuscript. Xuejun Gan was involved with the acquisition of data, drafting of the manuscript, and statistical analysis. Xiaojing Cheng was involved with basic study concept and design. Yongning Jia was involved with clinical study concept and design. Xiaohuan Tang and Gangjian Wang performed bioinformatics analysis, while Hong Du and Xiaomei Li provided assistance in experimental design. Xiaofang Xing was involved with IHC and critical revision of the manuscript for important intellectual content. Ziyu Li was involved with the study concept and design and obtained funding, technical support, and study supervision. All authors read and approved the final manuscript.

## CONFLICT OF INTEREST STATEMENT

The authors declare no conflict of interest.

## ETHICS STATEMENT AND CONSENT TO PARTICIPATE

The study was conducted in accordance with the Declaration of Helsinki, and approved by the Institutional Review Board of Peking University Cancer Hospital (2019KT10). Informed consent was obtained from all subjects involved in the study.

## Supporting information

Supporting Information

Supporting Information

Supporting Information

Supporting Information

## Data Availability

The gene expression data of public gastric cancer cohorts were obtained from the Cancer Genome Atlas (https://portal.gdc.cancer.gov/) and the Gene Expression Omnibus (GEO) database (https://www.ncbi.nlm.nih.gov/geo/) under accession numbers GSE84437. Individual participant data and other data supporting the findings of this study are available from the corresponding authors upon reasonable request.
