# Supplementary material for: ABCC2 induces metabolic vulnerability and cellular ferroptosis via enhanced glutathione efflux in gastric cancer
Source: Clin Transl Med. 2024 Aug 2;14(8):e1754. doi: 10.1002/ctm2.1754 (PMC11296884; doi:10.1002/ctm2.1754)
Supplement: Supplementary file 1 — Supporting Information [file CTM2-14-e1754-s004.docx]

**Supplementary Methods**

*Cell culture*

GC cells were cultured in high‐glucose Dulbecco's modified eagle media (DMEM, Gibco, Invitrogen Corporation, Carlsbad, CA, USA) supplemented with 10% fetal bovine serum (FBS, GIBCO, USA) or amino acid-deficient media (DMEM, Macgene, CM15022, Beijing, China) without FBS. All cells were incubated in a humidified atmosphere containing 5% CO2 at 37°C and grown for no more than 20 passages prior to experimentation. All of these cell lines were recently authenticated by STR profiling and tested for mycoplasma contamination.

*Establishment of stable cell lines*

Overexpression ABCC2 lentivirus was purchased from Genechem (Shanghai, China). AGS and MGC803 cells were transfected with ABCC2 lentiviruses or control for 48 h and then screened with 1μg/mL puromycin for 2 weeks to generate stable ABCC2‐overexpression or control cells. Stable ABCC2-knockout SGC7901 and BGC823 cells were established using CRISPR-Cas9 technology according to the manufacturer's instructions. The detailed sequences of the ABCC2 sgRNA were listed in Supplementary Table 2. An empty vector was applied as normal control (NC). After treatment with puromycin, monoclonal ABCC2-knockout cells were picked and expanded, and then ABCC2-knockout-cell clones were verified by western blotting and immunofluorescence.

*Western blotting*

Cells were lysed in RIPA lysis buffer (R0010, Beijing Solarbio Science & Technology, China) containing protease inhibitor cocktail (Roche, Basel, Switzerland) on ice for 15 min and then centrifuged at 15000×g for 10 minutes at 4°C. We collected the supernatants and quantified them utilizing the bicinchoninic acid kit. Equal amounts of protein were separated by 12% sodium dodecyl sulfate-polyacrylamide gel electrophoresis (SDS-PAGE) and transferred onto nitrocellulose (NC) membranes (HATF00010, Millipore, USA). After blocking with 5% skim milk for 1 hour at room temperature, the membrane was incubated with specific primary antibodies diluted at 4°C overnight, followed by the fluorescent-tagged secondary antibody incubation at room temperature for 1 hour. Finally, the protein bands were visualized using the Odyssey CLx system (Licor). The primary antibody against ABCC2 (ab998) was purchased from Abcam (Cambridge, MA, USA). The anti-GAPDH (#60008–1-Ig) was purchased from ProteinTech. Protein levels were normalized to GAPDH. All experiments were performed at least three times.

*Immunofluorescence (IF)*

Cells were seeded onto pre-prepared coverslips for 24 hours. After cell attachment, 4% paraformaldehyde was applied to fix cells for 15 min, followed by permeabilization with 0.1% Triton X-100 for 5 min and then blocked with 5% normal goat serum for 30 min. The cells were incubated with primary antibody against ABCC2 (Abcam, EPR10998, 1:1000) at 4°C overnight, then labeled with Alexa Fluor-594 conjugated anti-rabbit secondary antibody (1:200) for 1 hour at 37°C. Hoechst (#33342, Beyotime, China) was used as a nuclear counterstain. Images were captured with a Zeiss LSM700 confocal laser microscope (Leica, Germany).

*cell proliferation and migration assays in vitro*

IncuCyte Live-Cell Imaging Systems (Essen Bioscience, Ann Arbor, MI, USA) measured cell proliferation, a label-free, noninvasive assay for cellular confluence. Appropriate cell numbers were seeded into 96-well plates, and cell proliferation was assessed by collecting real-time data on cell confluence and cell viability. All experiments were performed three times.

For the migration ability detection, trans-well migration assay was conducted using 8 μm pore chambers (Corning Life Science, Woburn, MA, USA). 1 × 10^5^ cells suspended by 300 μl serum-free DMEM were seeded in the upper chamber and 800 μl DMEM pre-mixed with 10% FBS alone were added to the lower chamber. After incubation for 24 h, the non-migration cells on the upper surface of the filters were removed. Then, the filters were fixed in methanol, stained with 0.1% crystal violet, and quantified by counting ten randomly selected microscopic views using an inverted light microscope (magnification, 200×, Nikon Corporation, Japan). The experiments were performed in triplicate.

*OCR and ECAR rate measurements*

The Seahorse XF96 analyzer (Seahorse Bioscience, Agilent) was used to analyze the extracellular acidification rate (ECAR) and cellular oxygen consumption rate (OCR). ECAR measurements were performed by Seahorse XF Glycolysis Stress Test Kit (103020-100, Agilent Technologies, Palo Alto, CA, USA), and OCR measurements were performed by Seahorse XF Cell Mito Stress Test Kit (103015-100, Agilent Technologies, Palo Alto, CA, USA). Briefly, cells were seeded in a 96-well XF cell culture microplate in a growth medium for 24h before assay according to the manufacturer's protocols. The sensor cartridge was hydrated using a Seahorse XF Calibrant overnight at 37°C in a non-CO2 incubator. After baseline detections, the Seahorse automatically inserted various reagents in turn: 10 mM glucose, 1 μM oligomycin (the oxidative phosphorylation inhibitor), 50 mM 2‐DG (2‐deoxy‐D‐glucose, the glycolytic inhibitor) for ECAR, and 1 μM oligomycin, 1 μM FCCP (p‐trifluoromethoxy carbonyl cyanide phenylhydrazone, the reversible inhibitor of oxidative phosphorylation), 0.5 μM Rote/AA (rotenone plus the mitochondrial complex III inhibitor antimycin A, the mitochondrial complex I inhibitor) for OCAR. ECAR in mpH/min and OCR in pmol/min are reported by Seahorse XF96 Wave software.

*Glutathione (GSH) level detection*

BGC823 (5 × 10^4^ cells) and SGC7901 (2 × 10^4^ cells) were seeded in 6-well plates. After 12 h of incubation with different conditions (N: normal DMEM medium. NN: amino acid-free DMEM medium. Hank: Hank's solution. E: Erastin 10 μM), we harvested these cells. According to the manufacturer's instructions provided by the GSH and GSSG Assay Kit (Product No. S0053, Beyotime), we added these cells to the protein removal reagent solution followed by quick freeze-thaw cycles twice utilizing liquid nitrogen and 37°C water bath, and then stewing for 5 min at 4°C and centrifuged at 10,000 × g for 10 min. Next, we took the supernatant and detected the absorbance of each well at 410 nm through a microplate reader. Finally, calculate each group's GSH concentration and GSH/GSSG ratio according to the standard curve built via standard products.

*Lipid peroxides measurements*

Levels of intracellular Lipid peroxides were measured using the Spy-LHP analog Liperfluo (Product No. L248, Dojindo, Japan), which could be specifically oxidated by lipid peroxides, thereby producing strong fluorescence emission in organic solvents. First, appropriate cell numbers were seeded in 24-well plates. After cell attachment, the supernatant was discarded, and then the cells were washed with serum-free DMEM medium. Added the Liperfluo solution to each well at 1 μM, which was diluted in serum-free DMEM and incubated cells at 37°C for 30 min in the dark, followed by washing with 200μl HBSS solution twice. Finally, the fluorescence signals were captured by Zeiss LSM700 confocal laser microscope (emission wavelength: 525nm and excitation wavelength: 488nm) directly or directly examined by flow cytometry after the adherent cells were digested by trypsin.

*Transmission electron microscopy*

After centrifuge and precipitation, cells were collected and fixed with 3% glutaraldehyde in 0.1 M phosphate buffer (pH 7.4), followed by the fixation with 1% OsO4. Stain the specimen with uranyl acetate and plumbous nitrate in sequence. Then, prepare 60-80nm thick slices after dehydration and put the slice under a JEM-1230 transmission electron microscope (JEOL, Tokyo, Japan). Finally, high-resolution digital images were acquired from randomly selected five fields at each condition.

*Determination of cellular reactive oxygen species (ROS)*

Total intracellular ROS was determined by staining cells with dichlorofluorescin diacetate (DCFH-DA, Beyotime, Jiangsu, China). Gastric cancer cells were pretreated with different reagents (RSL3 0.5um; Fer-1 20um) under different nutrient conditions. Then, cells were washed with PBS and incubated with 10 μM DCFH-DA at 37°C for 30 min in the dark room. The relative ROS level of the cells was measured by flow cytometry (BD AccuriTM C6).

*Functional assay in vivo*

Before subcutaneous injection, all cells precultured with high‐glucose DMEM (Gibco, Invitrogen Corporation, Carlsbad, CA, USA) supplemented with 10% FBS or amino acid-deficient media (DMEM, Macgene, CM15022, Beijing, China) without FBS in a humidified atmosphere (5% CO2, 37°C) for 24 h. Then 1 × 10^7^ ABCC2-knockout SGC7901 cells and non-target (NC) SGC7901 cells were injected subcutaneously into the left and right flanks of NOD/SCID male mice (four-week-old, 18-20g), respectively. Tumor growth was monitored every 3 days by measuring the width and length of the tumors with calipers and visualized by a Xenogen IVIS imaging system (Caliper Life Sciences). Simultaneously, the mice were treated with various drugs (RSL3 100 mg/kg, FER-1 1mg/kg) by intraperitoneal injection every 3 days. The volumes of tumors were calculated using the following formula: V = (L × W^2^) × 0.5 (L represents the length and W represents the width of each tumor). 21 days later, the mice were euthanized with carbon dioxide, and the subcutaneous tumors were harvested and weighed immediately. The ethics committee of Peking University Cancer Hospital and Institute approved animal experiments in this study, and our performance abided by the national and institutional guidelines.

*WES sequencing and data pre-processing*

DNA from tumor and adjacent normal (paracarcinoma) tissues was extracted using the QIAamp DNA FFPE Tissue Kit (Qiagen, USA). DNA was quantified using the Qubit 2.0 Fluorometer (Life Technologies, USA), and quality was assessed with the Bioanalyzer 2100 (Agilent Technologies, USA). The genomic DNA was sheared into approximately 250-bp fragments using the M220 Focused-ultrasonicator (Covaris, USA). A genomic library was then constructed using the KAPA Hyper Prep Kit (KAPA Biosystems, USA). Following the manufacturer's protocol, the whole-exome capture was performed with the xGen™ Exome Hybridization Panel (Integrated DNA Technologies, USA). The enriched libraries were amplified using Illumina p5 and p7 primers in KAPA HiFi HotStart ReadyMix and purified with Agencourt AMPure XP beads. Sequencing was conducted on the Illumina HiSeq 4000 platform, producing paired-end 150-bp reads per the manufacturer's instructions. Whole-exome sequencing achieved median depths of 224.1× for tumor tissues and 117.8× for matched normal samples.

*RNA sequencing (RNA-seq) of tissue samples*

Total RNA was isolated using SMART-Seq® HT Kit. Paired-end libraries were synthesized by using the TruSeq® RNA Sample Preparation Kit (Illumina, USA) following TruSeq® RNA Sample Preparation Guide. Briefly, the poly-A containing mRNA molecules were purified using poly-T oligo-attached magnetic beads. Following purification, the mRNA is fragmented into small pieces using divalent cations under 94℃ for 8 min. The cleaved RNA fragments are copied into first strand cDNA using reverse transcriptase and random primers. This is followed by second strand cDNA synthesis using DNA Polymerase I and RNase H. These cDNA fragments then go through an end repair process, the addition of a single ‘A’ base, and then ligation of the adapters. The products are then purified and enriched with PCR to create the final cDNA library. Purified libraries were quantified by Qubit® 2.0 Fluorometer (Life Technologies, USA) and validated by Agilent 2100 bioanalyzer (Agilent Technologies, USA) to confirm the insert size and calculate the mole concentration. Cluster was generated by cBot with the library diluted to 10 pM and then were sequenced on the Illumina HiSeq Xten (Illumina, USA). The library construction and sequencing were performed at Shanghai Biotechnology Corporation.

*Metabolic profiling.*

To elucidate central carbon metabolism, targeted LC/MS-MS analysis was conducted. Control (NC) and ABCC2-knockout (KO) cells were cultured for 12 hours in either amino acid-free medium or standard DMEM (NC group). Metabolites were extracted using 80% methanol at dry ice temperatures. The extracts were then analyzed using a Shimadzu LC Nexera X2 UHPLC system coupled with a QTRAP 5500 LC-MS/MS (AB Sciex). Chromatographic separation was achieved using an ACQUITY UPLC BEH Amide analytical column. The mobile phase consisted of buffer A (10 mM ammonium acetate in water, pH 8.8) and buffer B (10 mM ammonium acetate in acetonitrile/water (95/5), pH 8.2). The gradient elution profile was as follows: 95–61% buffer B over 7 minutes, 61–44% buffer B over 9 minutes, 61–27% buffer B over 0.2 minutes, and 27–95% buffer B over 0.8 minutes. The column was re-equilibrated with 95% buffer B at the end of the run. 13C‐nicotinic acid (Toronto Research Chemicals) served as the internal standard. MultiQuant 3.0.2 software (AB Sciex) integrated the extracted MRM peaks.

*Droplet digital polymerase Chain reaction (ddPCR) detection*

The sequence of the used primers and probes for ddPCR are listed as follows:

| Primer/Probe Name | Sequence | Reporter |
| --- | --- | --- |
| rs717620-F | AAATGGTTGGGATGAAAGGTCA | / |
| rs717620-R | ATGATTCCTGGACTGCGTCTG | / |
| rs717620-WT | AGAGTCTTCGTTCCAG | 5'VIC |
| rs717620-MU | AGAAGAGTCTTTGTTCC | 5'FAM |

Cycling conditions for the reaction were 95°C for 10 min, followed by 40 cycles of 94°C for 30 sec and 60°C for 1 min, then 98°C for 10 minutes, and finally a 16°C hold on a Life Technologies Veriti thermal cycler. Data were analyzed using QuantaSoft v1.7.4 (Bio-Rad Laboratories, USA). The 300 bp synthetic mutant DNA fragment (ABCC2 rs717620 C>T mutation [ABCC2-MU]) and 300 bp synthetic wildtype DNA fragment (human genomic DNA [ABCC2-WT]) were constructed by Sangon Biotech (Shanghai) Co., Ltd. (Shanghai, China) and used as positive control and negative control, respectively. In addition, DNase/RNase free water was used as no template control.

*ABCC2 CRISPR/Cas9 KO stable clone*

sgRNAs for SpCas9-mediated genome editing were designed using the CHOPCHOP web-based sgRNA design tool [1]. Two sgRNA guide sequences were selected for subsequent validation based on the highest cutting efficiency and lowest off-target possibility. The detailed sequences of the ABCC2 sgRNA were listed in Supplementary Table 2. Through inserting the sgRNA sequence into the LentiCRISPRv2 vector, the ABCC2-knockout lentiviral expression vector was constructed. Then, lentiviral particles were produced by co-transfecting HEK293T cells with the ABCC2-knockout plasmid together with packaging plasmids pLP1, pLP2, pLP/VSVG using Lipofectamin 2000 (Thermo Fisher Scientific) following the manufacturer’s instructions. When the confluency reached 60%, tumor cells were infected with viral supernatant for 3 days. Following by antibiotics selection with 2 μg/ml puromycin, single clone was selected by serial dilution in 96-well platform. After expansion of puromycin-positive clones, the genomic DNA and protein were extracted from each stable clones and deletion efficiency was detected by PCR and Western Blotting.

*Tissue dissociation and organoid culturing*

Fresh gastric cancer tissues were carefully minced into small pieces using fine dissection scissors. The fragments were then processed using a tumor dissociation kit (Miltenyi Biotec) according to the manufacturer's instructions, with an incubation period of 60 minutes at 37°C. To stop the digestion, DMEM containing 10% FBS was added. The resulting cell suspension was passed through a 70 μm nylon cell strainer and centrifuged at 350 g for 5 minutes. Following centrifugation, the RBC lysis buffer was removed, and the cells were mixed with 50% growth factor-reduced Matrigel (Corning) before being seeded into ultralow-attachment 24-well plates. Once the Matrigel solidified after 30 minutes, warm organoid isolation culture medium was added.

The culture medium was comprised of Advanced DMEM/F12 supplemented with 1% penicillin/streptomycin, 1% glutamax, 10-mM HEPES, 1:50 B27 supplement (without vitamin A), 1:100 N2 supplement, 1.25 mM N-acetyl-L-cysteine (Sigma), 10-mM nicotinamide, 10-nM recombinant human (Leu15)-gastrin I, 50 ng/mL recombinant human EGF, 100 ng/mL recombinant human FGF10, 25 ng/mL recombinant human HGF, 10 μM forskolin, 5-μM A83-01, 10 μM Y27632 (Sigma), 25 ng/mL recombinant human Noggin, 500 ng/mL Rspo-1 and 100 ng/mL Wnt3a. The medium was refreshed with new media every 2-3 days. Organoid cultures were passaged biweekly, either through mechanical dissociation or by using 0.25% Trypsin-EDTA to fragment them into smaller pieces. The cells were treated with various reagents (RSL3 at 10 μM; Fer-1 at 10 μM) for 72 hours, after which cell viability was measured using the CCK-8 assay (Dojindo, Japan).

*Bioinformatics analysis*

Public transcriptome sequencing data and survival data for GC patients were downloaded from the Gene Expression Omnibus (GEO) database (GSE84437) and The Cancer Genome Atlas (TCGA) database (https://cancergenome.nih.gov/). All statistical analyses were performed utilizing R software (v4.0.2). Kaplan‐Meier curves were constructed to draw survival curves, and the log‐rank test was used to compare survival rates between groups. The differentially expressed genes (DEGs) between different groups were characterized with inclusion standard (|fold-change| > 1, adjusted P < 0.05) by utilizing the "limma" R package. Gene Ontology (GO) and Kyoto Encyclopedia of Genes and Genomes (KEGG) analyses were conducted according to the DEGs utilizing the "clusterProfiler" R package with a screening threshold (P < 0.05). Gene set enrichment analysis (GSEA) was performed to investigate the differences between different groups in signal pathways based on the hallmark gene set (h.all.v7.2) extracted from MSigDB database using GSEA software (v4.0.3; https://www.gsea-msigdb.org/gsea/index.jsp). A waterfall plot was performed to visualize and summarize the mutated genes of tissue in gastric cancer patients.
