# Supplementary material for: ABCC2 induces metabolic vulnerability and cellular ferroptosis via enhanced glutathione efflux in gastric cancer
Source: Clin Transl Med. 2024 Aug 2;14(8):e1754. doi: 10.1002/ctm2.1754 (PMC11296884; doi:10.1002/ctm2.1754)
Supplement: Supplementary file 2 — Supporting Information [file CTM2-14-e1754-s001.docx]

Supplementary tables

**Table S1. SNPs related to platinum/5-Fu-based drug resistance**

| **SNP** | **CHR** | **Position** | **Alleles** | **Gene^a^** | **Amino Acid Translation** | **Genotype** |
| --- | --- | --- | --- | --- | --- | --- |
| rs1801131 | 1 | 11794419 | C/A | *MTHFR* | Glu347Ala | 2/17/84 |
| rs1801133 | 1 | 11796321 | C/T | *MTHFR* | Ala140Val | 16/50/37 |
| rs1801159 | 1 | 97515839 | G/A | *DPYD* | Ile543Val | 8/42/53 |
| rs1801265 | 1 | 97883329 | C/T | *DPYD* | Cys29Arg | 3/15/85 |
| rs1801160 | 1 | 97770920 | A/G | *DPYD* | Val732Ile | 0/2/101 |
| rs17376848 | 1 | 97915624 | G/A | *DPYD* | Phe632Phe | 5/12/86 |
| rs2297595 | 1 | 98165091 | G/A | *DPYD* | Met166Val | 0/4/99 |
| rs1801019 | 3 | 124456742 | C/G | *UMPS* | Gly213Ala | 4/28/71 |
| rs1045642 | 7 | 87138645 | T/C | *ABCB1* | Ile1145Ile | 18/48/36 |
| rs717620 | 10 | 101542578 | T/C | *ABCC2* | 5′UTR | 3/36/64 |
| rs3212986 | 11 | 45912736 | T/G | *ERCC1* | Gln504Lys | 12/48/41 |
| rs1695 | 11 | 67352689 | G/A | *GSTP1* | Ile105Val | 1/57/45 |
| rs25487 | 19 | 44055726 | A/G | *XRCC1* | Gln399Arg | 9/33/61 |

a. MTHFR (methylenetetrahydrofolate reductase); MTHFR, DPYD (dihydropyrimidine dehydrogenase), UMPS (uridine monophosphate synthetase), ABCB1 (ATP binding cassette subfamily B member 1), ABCC2, ERCC1 (excision repair cross-complementing 1), GSTP1 (glutathione S-transferase pi 1), and XRCC1 (X-ray repair complementing defective repair in Chinese hamster cells 1).

**Table S2. The Oligo sequences of ABCC2-sgRNA**

| **sgRNA** | **Oligo nucleotide sequence (5’→3’)** |
| --- | --- |
| sgRNA1 | Forward: GACCGCACTTCAGCGAGACCGTATC |
|  | Reverse: AAACGATACGGTCTCGCTGAAGTGC |
| sgRNA2 | Forward: GACCGTGGTCCTGGATTTATACACG |
|  | Reverse: AAACCGTGTATAAATCCAGGACCAC |


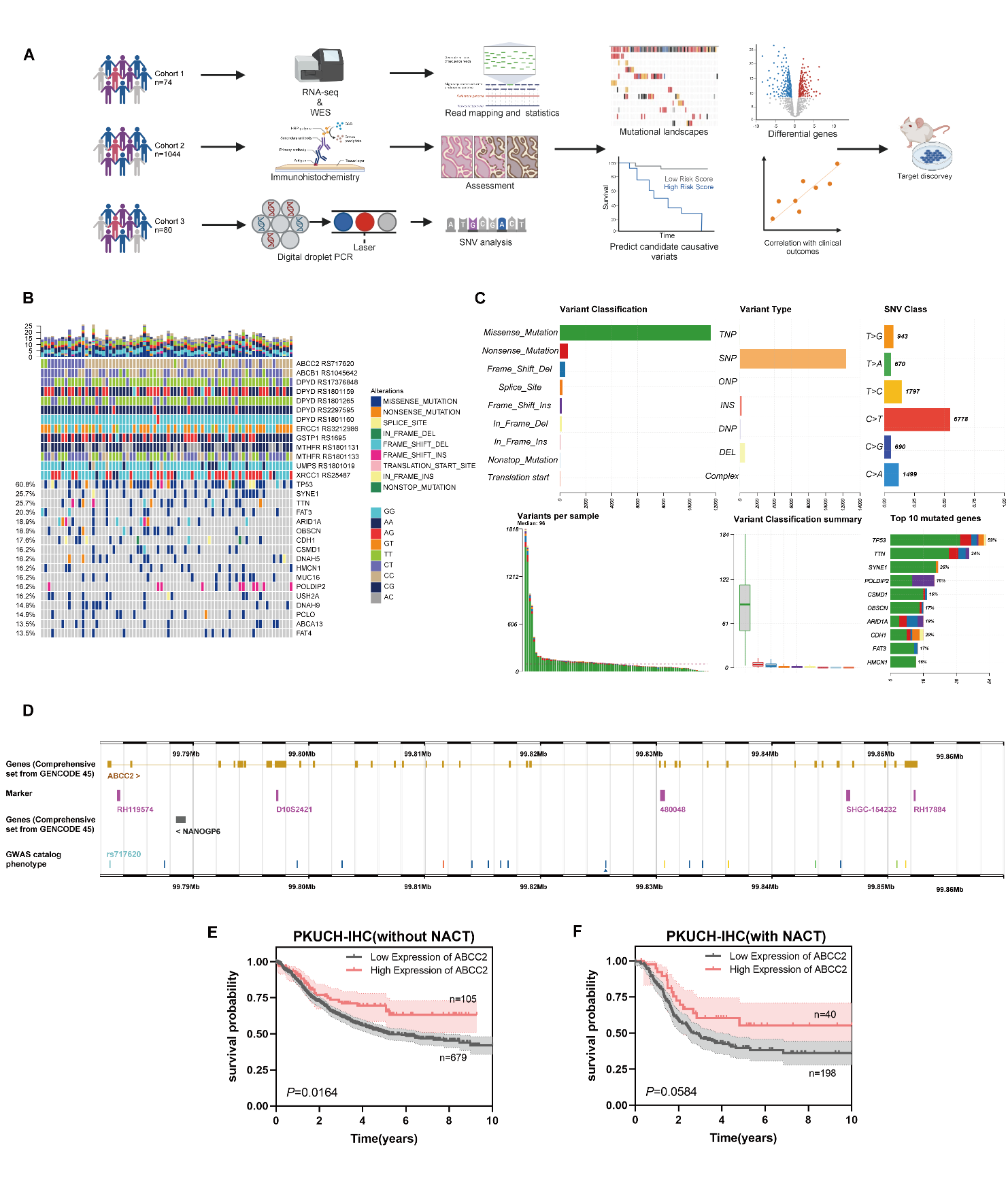
Supplementary figures

**Figure S1. Analysis of gastric cancer WES data.**

1. Overview of the study populations (cohorts) and schematic workflow.
2. Molecular map of tissue in gastric cancer patients from PKUCH cohort. The graph's body presents information about each gene, encompassing various mutation types across different samples, along with the overall mutation frequency among patients with available whole-exome sequencing (WES) samples.
3. The cohort summary plot shows the distribution of variants according to variant classification, type, and SNV class.
4. The structure of ABCC2 gene contained regions of rs717620 and markers.
5.
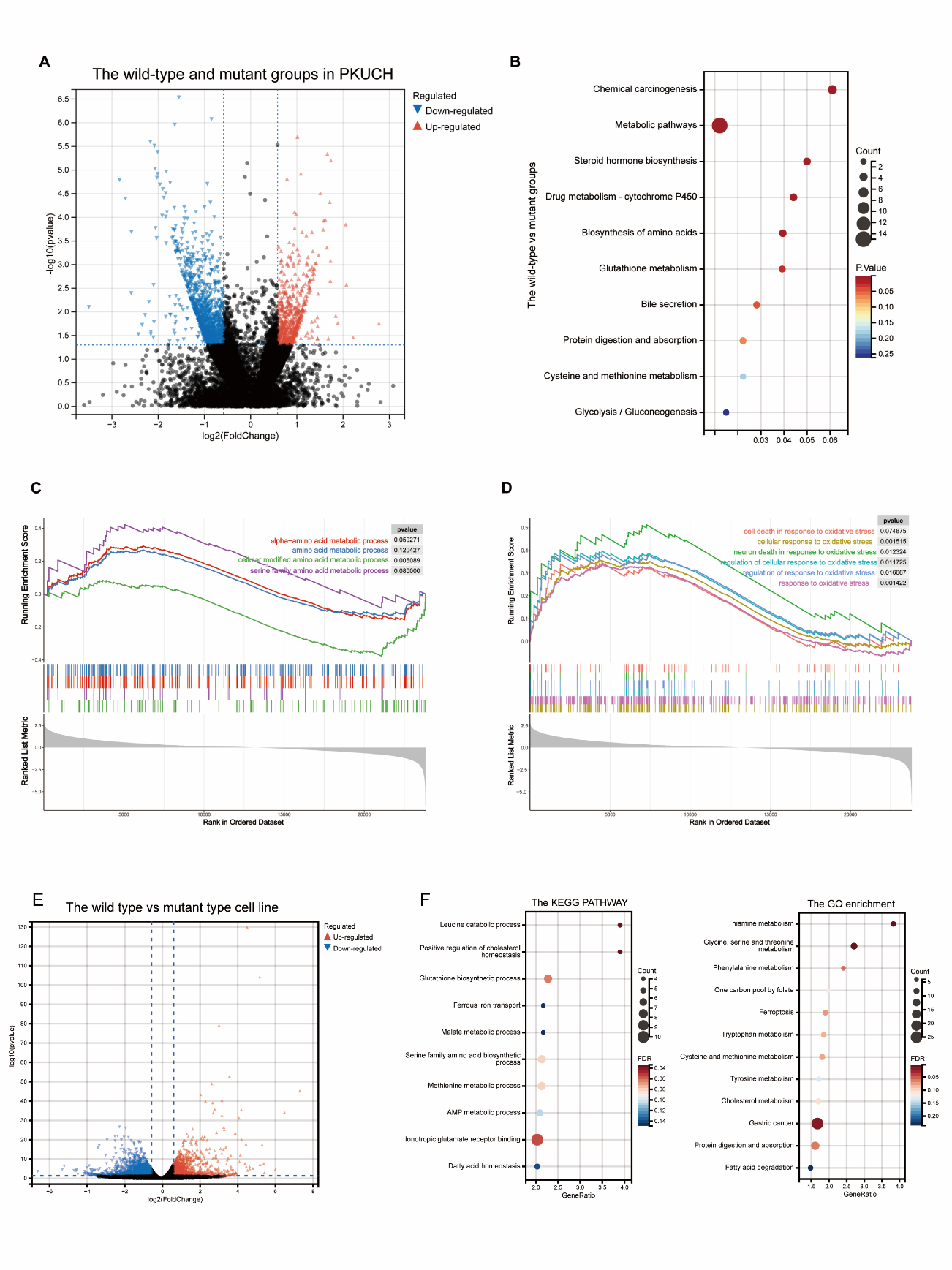
Kaplan-Meier survival analysis of OS according to ABCC2 levels in 784 GC patients who did not receive neoadjuvant therapy for gastric cancer and 238 GC patients who receive neoadjuvant therapy for gastric cancer.

**Figure S2. Analysis of gastric cancer RNA-seq data.**

1. Volcano plots showing differentially expressed genes between the wild-type (CC) and mutant (CT/TT) groups in PKUCH, using Log2(Fold Change) differences versus–Log10 (*P*-value) for indicated comparisons. Genes exhibiting FC > 1.5 and *P-*value < 0.05 are colored.
2. KEGG pathway enrichment results of differential gene expression in ABCC2-CC and CT/TT genotype patients.

(C&D) Gene Set Enrichment Analysis demonstrating that amino acid metabolism and oxidative stress-related genes were significantly enriched in ABCC2- CT/TT genotype patients.

1. Volcano plots showing differentially expressed genes between the wild-type and mutant cell line, using Log2(Fold Change) differences versus–Log10 (*P*-value) for indicated comparisons. Genes exhibiting FC > 1.5 and *P-*value < 0.05 are colored.
2. KEGG and GO pathway enrichment results of differential gene expression in ABCC2-WT and mutant cell line,


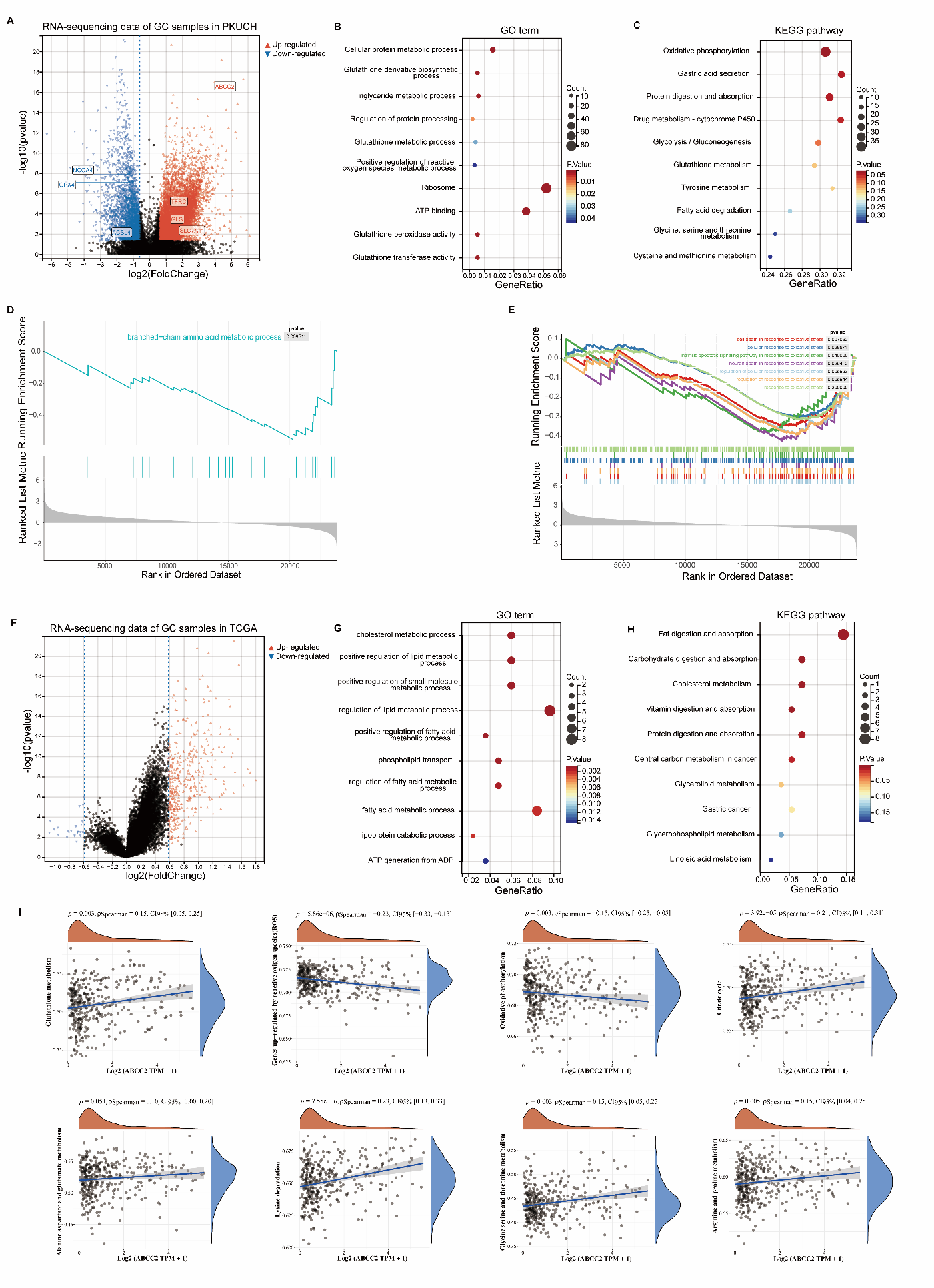


**Figure S3. Comprehensive RNA-sequencing analyses of GC samples in PKUCH and TCGA revealed that ABCC2 plays a crucial role in tumor metabolism and oxidative stress.**

(A) Volcano plots showing differentially expressed genes of GC samples in PKUCH, using Log2(Fold Change) differences versus–Log10 (*P*-value) for indicated comparisons. Genes exhibiting FC > 1.5 and *P-*value < 0.05 are colored.

(B&C) The GO and KEGG enrichment were employed to elucidate the primary biological actions of major potential mRNA. The abscissa indicates gene ratio, and the enriched pathways were presented in the ordinate. Colors represent the significance of differential enrichment, and the size of the circles represents the number of genes.

(D&E) Gene Set Enrichment Analysis demonstrating that amino acid metabolism and oxidative stress-related genes were significantly enriched in ABCC2-high expressed patients from PKUCH.

(F)  Volcano plots showing differentially expressed genes in TCGA-STAD cohort.

(G&H) The GO and KEGG enrichment were employed to elucidate the primary biological actions of major potential mRNA in TCGA-STAD cohort.

(I) Spearman correlation analysis revealed the ABCC2 expression was significantly correlated with ROS regulation and multiple amino acid metabolic pathways in TCGA-STAD cohort. The abscissa represents the distribution of the gene expression, and the ordinate represents the distribution of the pathway score. The density curve on the right represents the trend in the distribution of pathway score, and the upper-density curve represents the trend in the distribution of the gene expression.


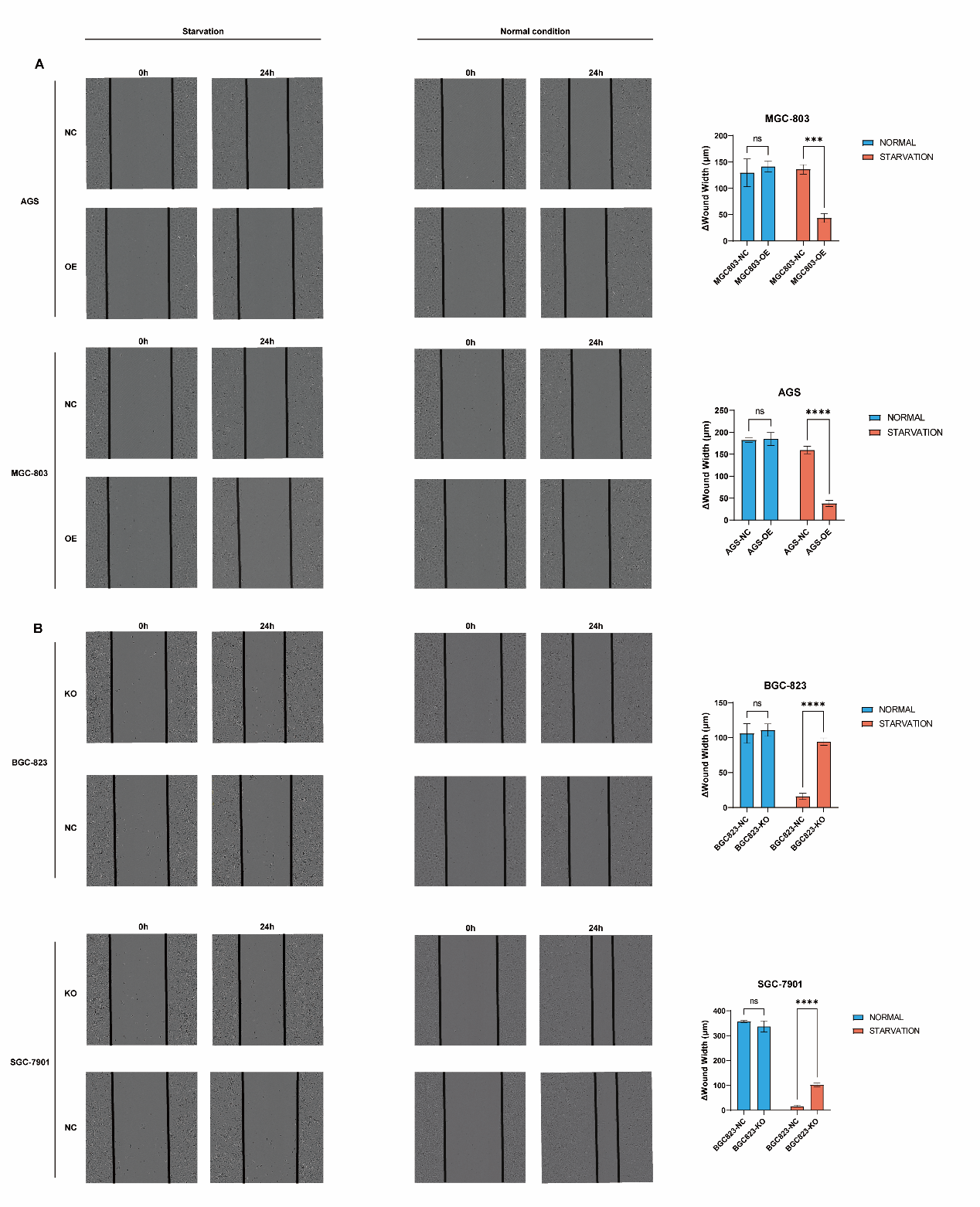


**Figure S4. Starvation impairs the migration capacity of ABCC2 high-expression cell lines.**

1. Scratch wound healing assays were performed to examine the cell migration in ABCC2 overexpressed MGC-803 and AGS cells.
2. Scratch wound healing assays were performed to examine the cell migration in ABCC2- knockout BGC-823 and SGC-7901 cells.

**
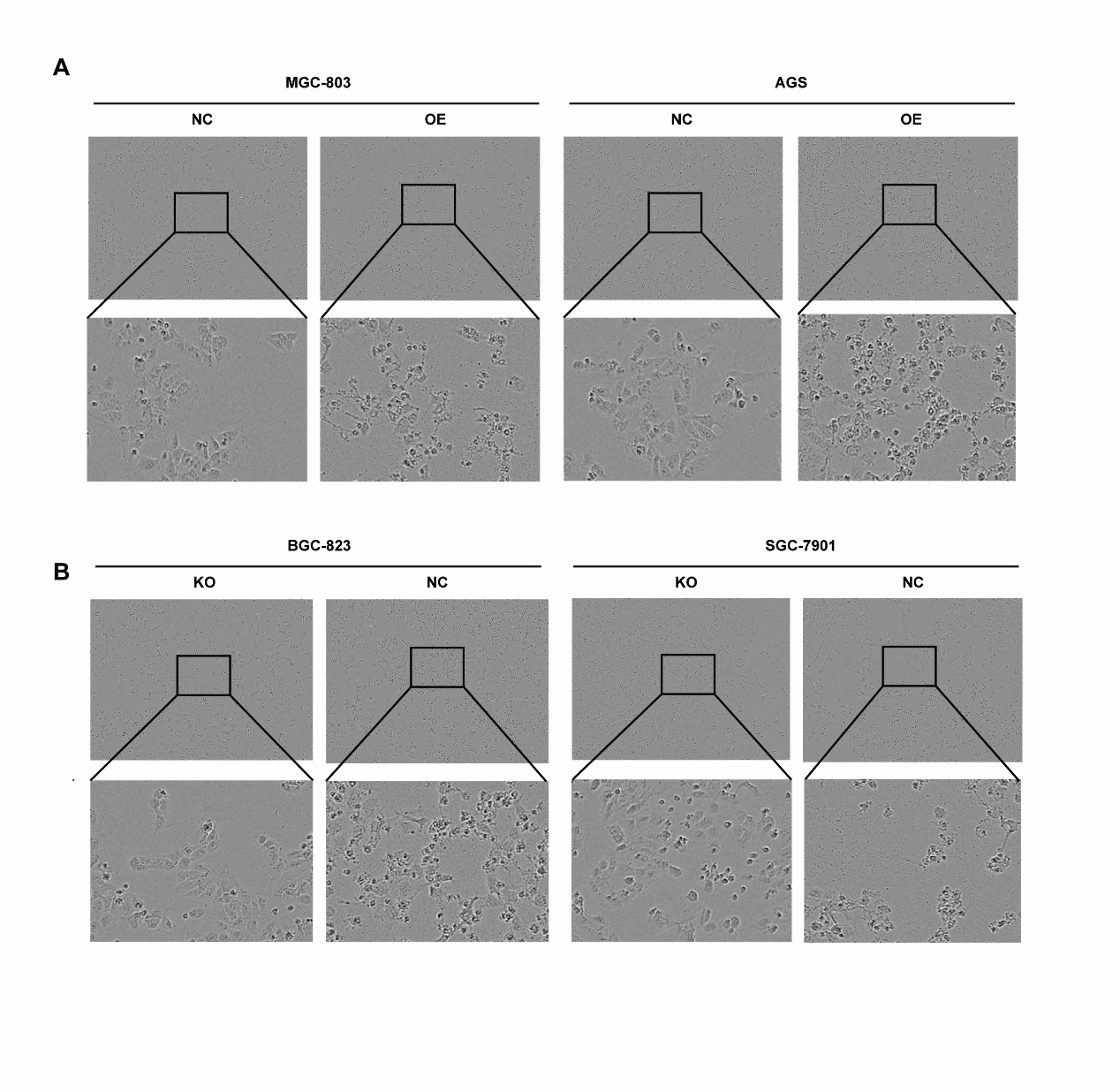
Figure S5. Starvation impairs the cell viability of ABCC2 high-expression cell lines.**

1. Representative cell images were captured using IncuCyte ZOOM to assess cell viability after 24 hours of amino acid starvation in ABCC2 overexpressed MGC-803 and AGS cells.
2. Representative cell images were captured using IncuCyte ZOOM to assess cell viability after 24 hours of amino acid starvation in ABCC2-knockout BGC-823 and SGC-7901 cells.

**
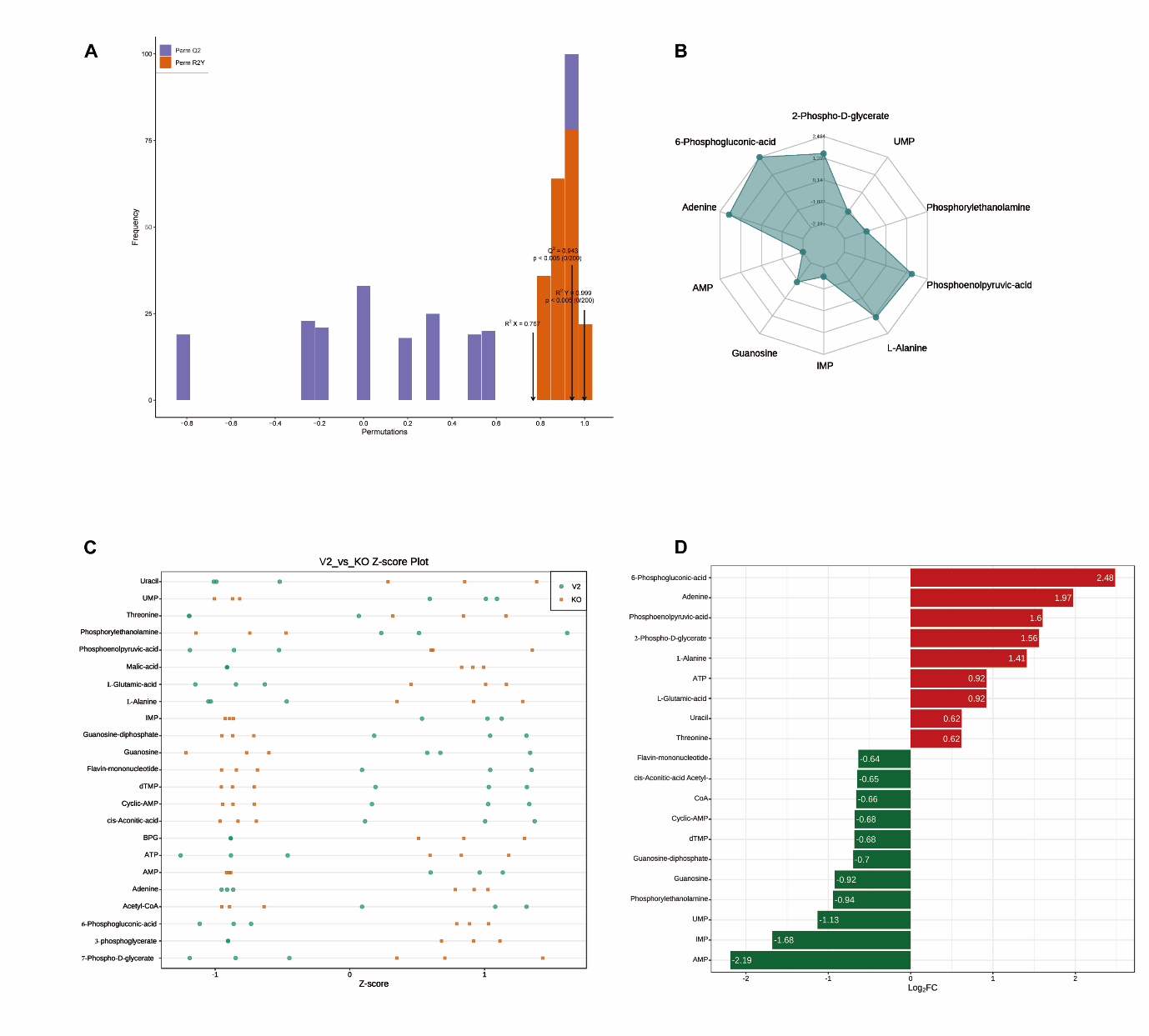
**

**Figure S6. ABCC2 triggers metabolic vulnerability and hinders cellular oxidative phosphorylation (OXPHOS) under starvation conditions.**

1. OPLS-DA model validation. The horizontal axis represents the model accuracy, and the vertical axis represents the frequency of the model's classification performance.
2. Differential metabolite radar chart analysis. The top 10 metabolites with the highest fold change (FC) values were chosen from the differentially identified metabolites, guided by specific filtering criteria, to construct the radar chart. Grid lines in the chart correspond to the magnitude of differential fold changes, and the green shadows are created by connecting lines that represent the differential fold changes of each substance.
3. Differential metabolite Z-score plot. The horizontal axis represents normalized Z-scores, the vertical axis represents differential metabolites, and differently colored points represent samples from different groups.
4. Differential metabolite bar plot. The horizontal axis represents the log2FC of differential metabolites, and the vertical axis represents the differential metabolites.


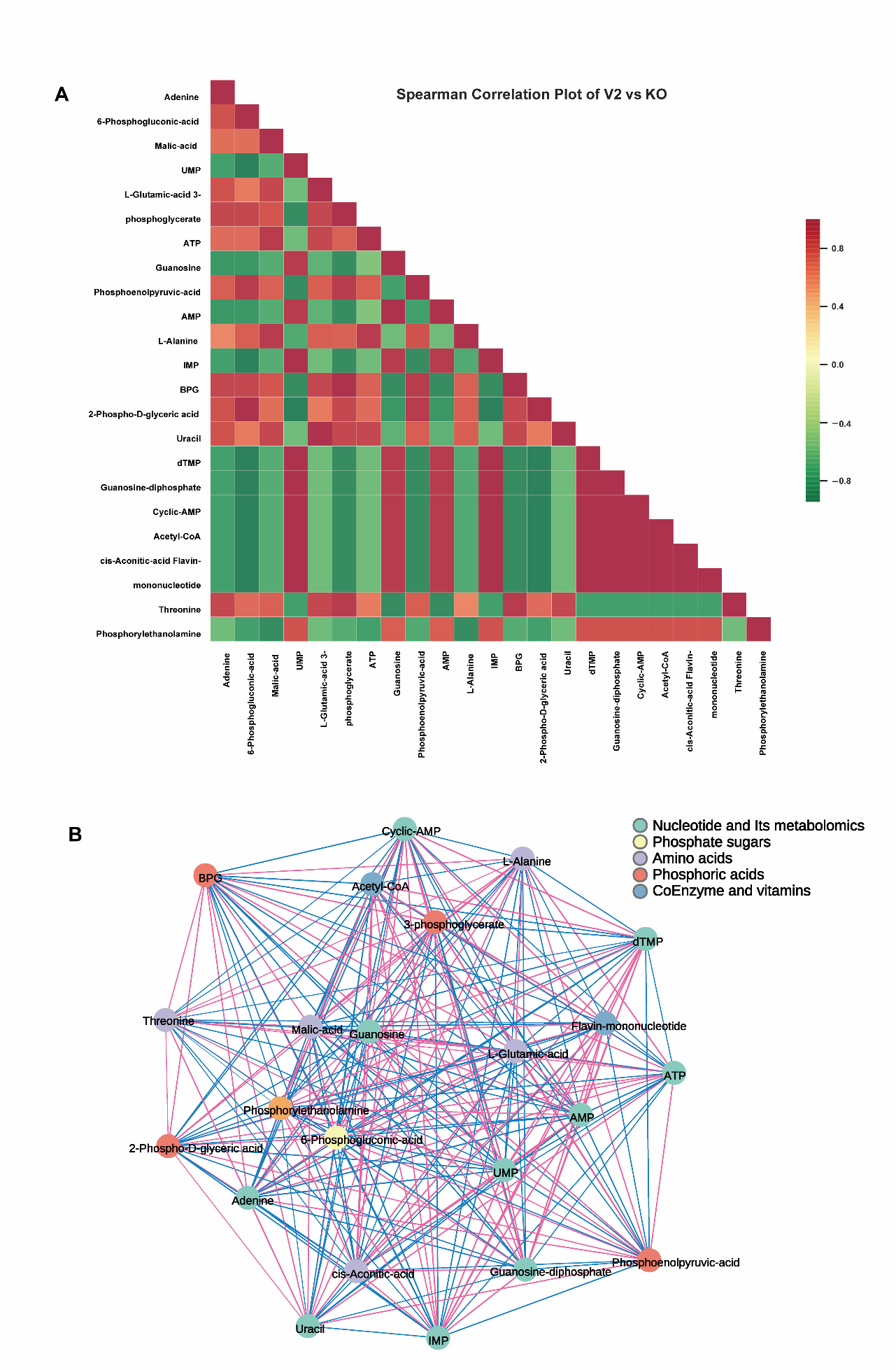


**Figure S7. Differential metabolite correlation analysis reveals the metabolic proximities**

1. Pearson correlation analysis is employed to assess the interrelation of differentially significant metabolites based on screening criteria.
2. Differential metabolite correlation network diagram. The dots in the graph represent significantly different metabolites, with dot size indicating the degree of connectivity; larger dots indicate greater connectivity. Red lines represent positive correlations, while blue lines represent negative correlations. The thickness of the lines corresponds to the absolute value of the correlation coefficient; thicker lines indicate stronger correlations.


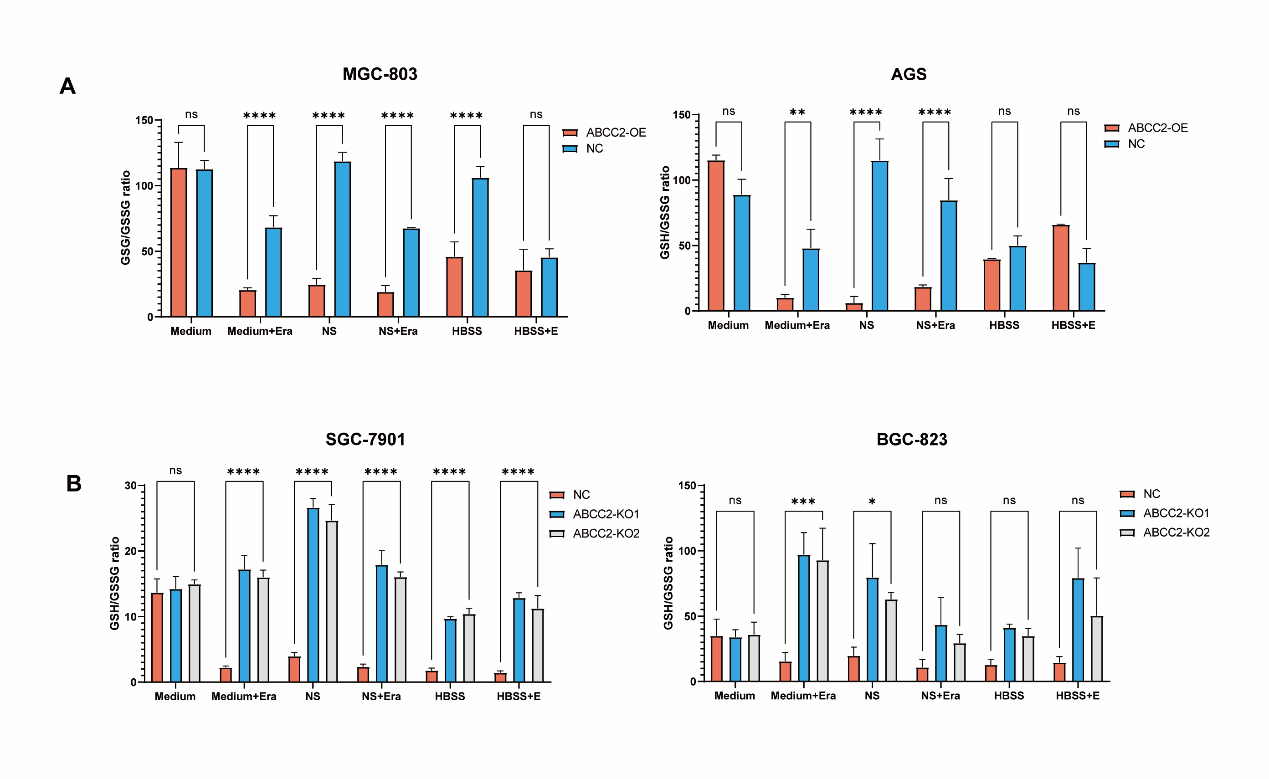


**Figure S8. ABCC2 induces a significant reduction in cellular amino acid levels through the extrusion of glutathione (GSH)**

1. GSH/GSSG ratio detected by Glutathione Assay Kit in ABCC2 overexpressed MGC-803 and AGS cells.
2. GSH/GSSG ratio detected by Glutathione Assay Kit in ABCC2-knockout BGC-823 and SGC-7901 cells.


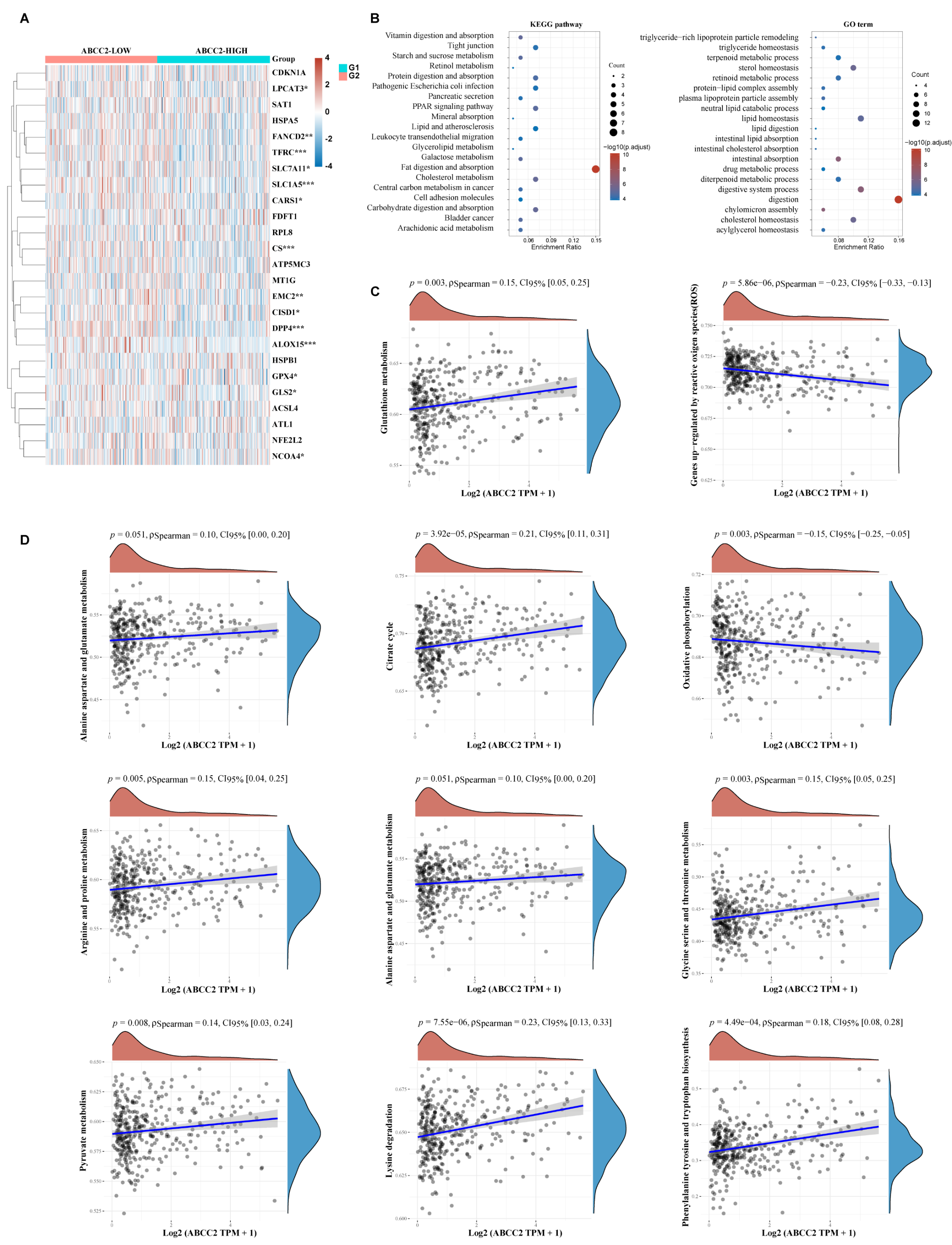


**Figure S9. Analysis of gastric cancer RNAseq data from the TCGA public database.**

(A) The heatmap of ferroptosis-related gene expression. The different colors represent the trend of gene expression in different samples. **P* < 0.05, ***P*< 0.01,****P*< 0.001, asterisks (*) stand for significance levels. The statistical difference between the two groups was compared through the Wilcox test, and the significance difference between the three groups was tested with the Kruskal-Wallis test.


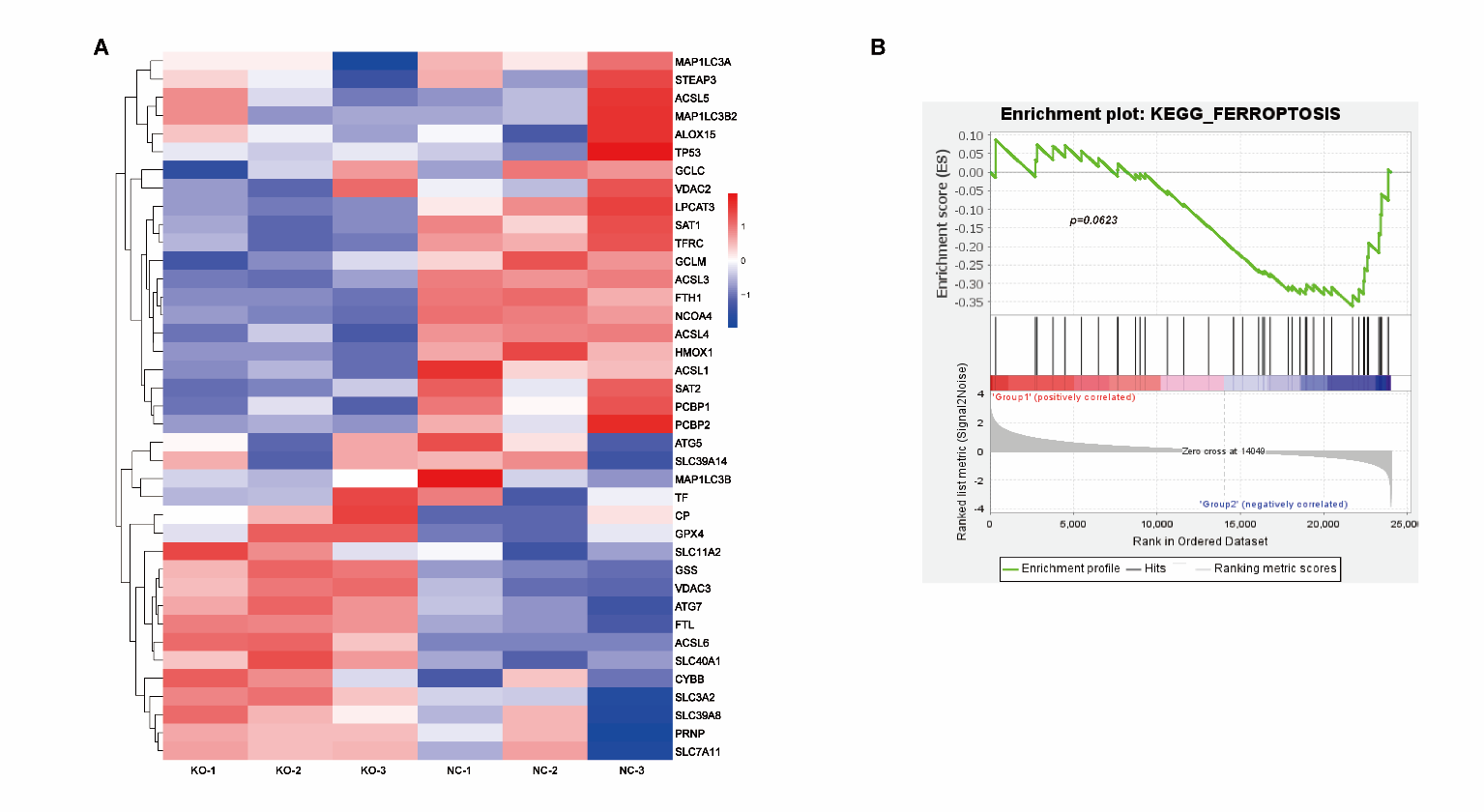


**Figure S10. ABCC2 deficiency induces ferroptosis in gastric cancer cells under amino acid starvation.**

1. The heatmap of ferroptosis-related gene expression.
2. GSEA analysis of differential gene expression in ABCC2 knockout SGC 7901 cells.


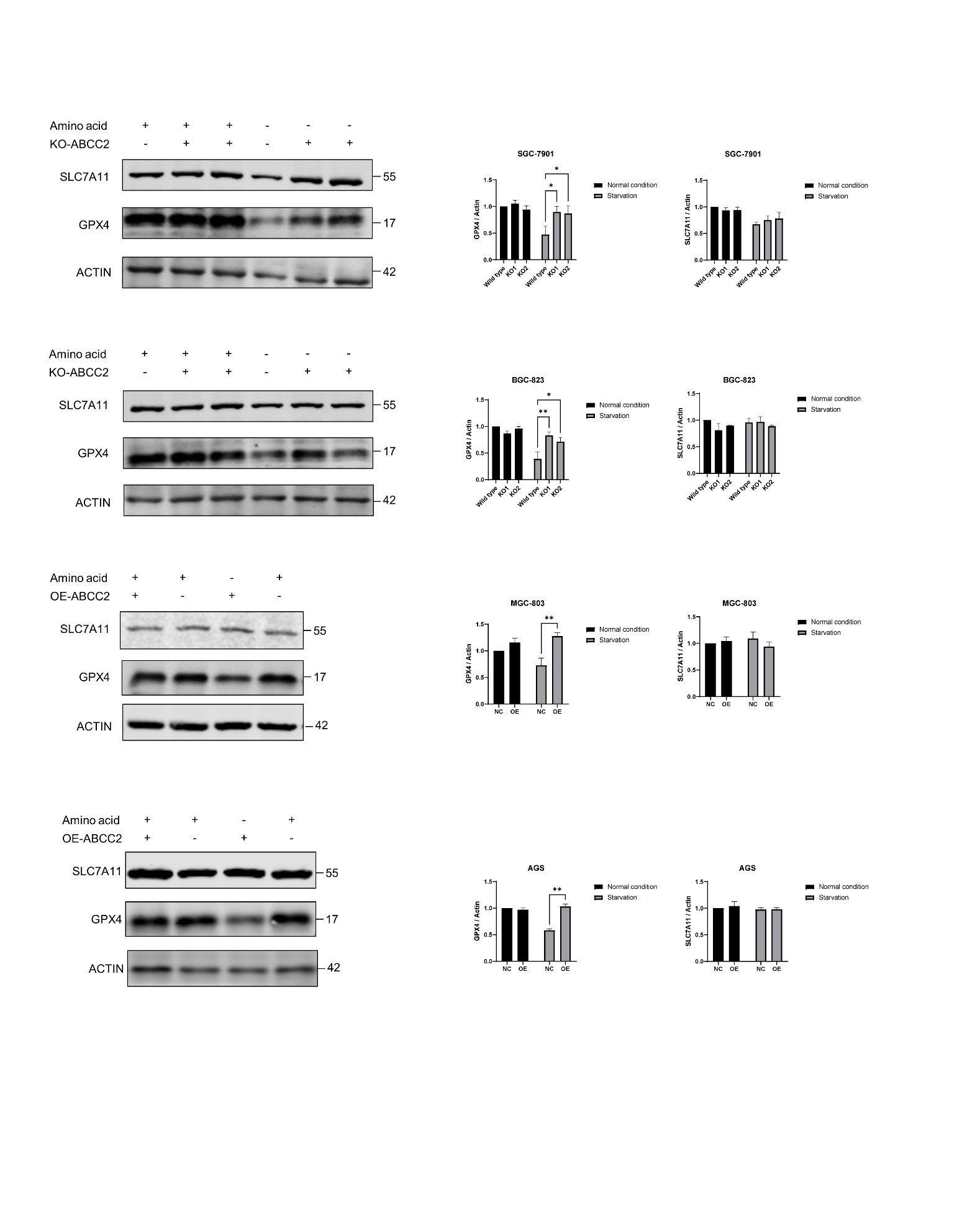
**Figure S11. The correlation between ABCC2 expression and SLC7A11 and GPX4**

Western blotting experiments to investigate the correlation between ABCC2 expression and SLC7A11 and GPX4 in four gastric cancer cell lines under different nutritional conditions.

**
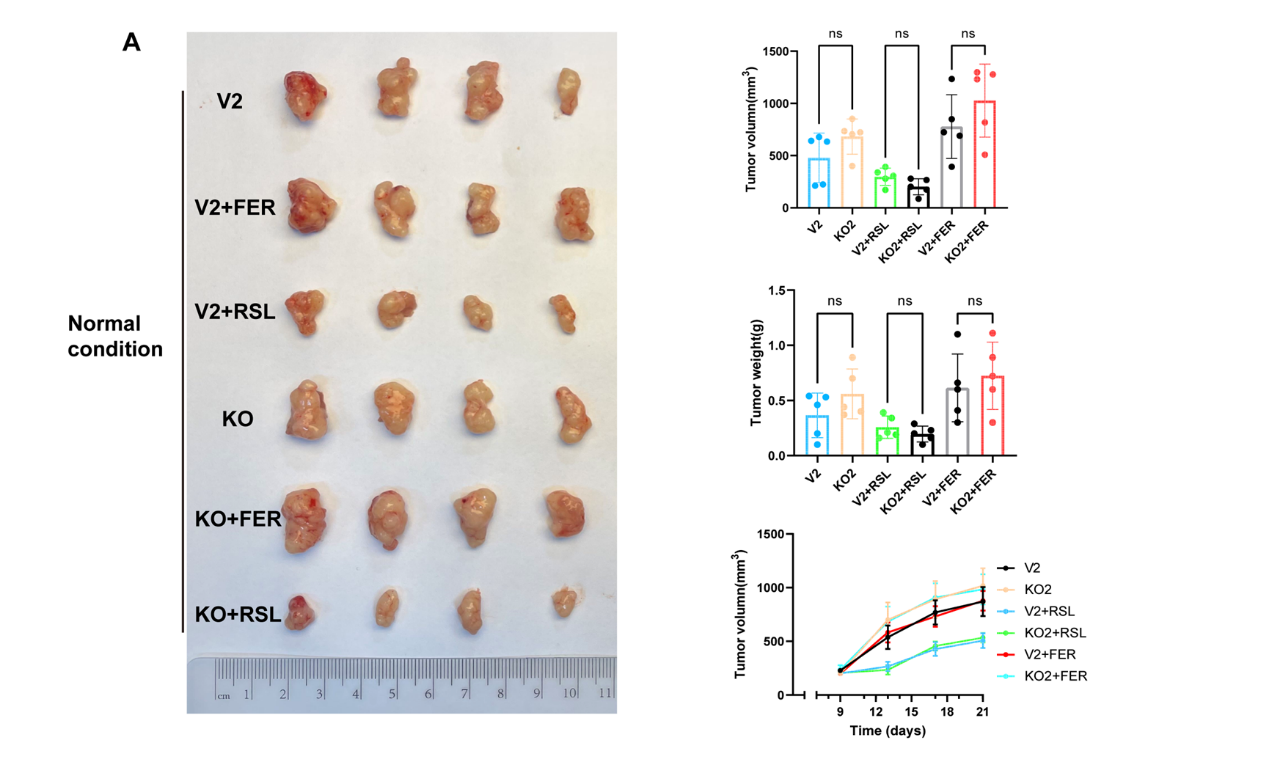
Figure S12. ABCC knockout gastric cancer cell line was significantly sensitive to ferroptosis inducers in vivo.**


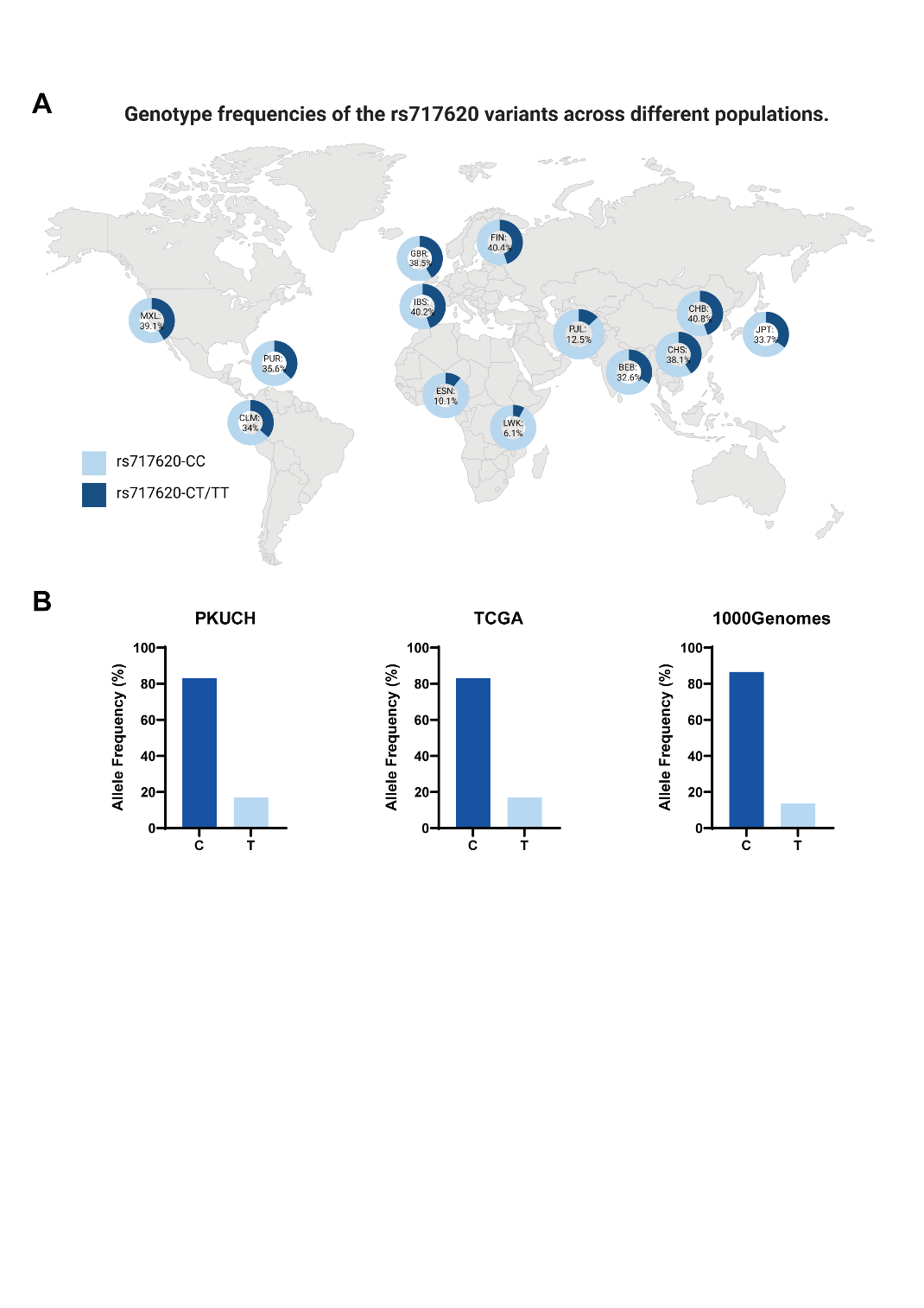
(A) The representative images and the quantification of xenograft in SGC7901 cells upon NT or ABCC2-KO (in normal medium) were implanted subcutaneously in NOD-SCID mice (n = 4). Tumor volumes were calculated after injection every 3 d for 21 d. Tumor weights are represented as mean ± SD.

**Figure S13. Genotype frequencies of the rs717620 variants across different populations.**

(A) Genotype frequencies of the rs717620 CT/TT variants obtained from the 1000 Genomes Project. The pie charts indicate the frequencies of the wild type (CC, dark blue) and mutant (CT / TT, light blue) alleles in each population sampled. The populations include: (1) Esan in Nigeria (ESN); (2) Luhya in Webuye, Kenya (LWK); (3) Colombian in Medellin, Colombia (CLM); (4) Mexican Ancestry in Los Angeles, California (MXL); (5) Puerto Rican in Puerto Rico (PUR); (6) Han Chinese in Bejing, China (CHB); (7) Southern Han Chinese, China (CHS); (9) Japanese in Tokyo, Japan (JPT); (8) Finnish in Finland (FIN); (9) British in England and Scotland (GBR); (10) Liberian populations in Spain (LBS); (11) Bengali in Bangladesh (BEB); (12) Punjabi in Lahore, Pakistan (PJL).

(B) The allele frequency of the rs717620 in the PKUCH, TCGA and 1000Genomes database.
